# Youth Correlates of Genetic Liability to Substance Use Disorders

**DOI:** 10.1016/j.bpsgos.2026.100835

**Published:** 2026-09-28

**Authors:** Sarah E. Paul, Aaron J. Gorelik, Nicole R. Karcher, Alex P. Miller, David A.A. Baranger, Emma C. Johnson, Kimberly H. LeBlanc, Gayathri J. Dowling, Deanna M. Barch, Alexander S. Hatoum, Arpana Agrawal, Ryan Bogdan

**Affiliations:** a Psychological and Brain Sciences, Washington University in St. Louis, St. Louis, Missouri; b Department of Psychiatry, Washington University School of Medicine, St. Louis, Missouri; c Department of Psychiatry, Indiana University School of Medicine, Indianapolis, Indiana; d Department of Psychiatry, Wisconsin College of Medicine, Milwaukee, Wisconsin; e Division of Extramural Research, National Institute on Drug Abuse, Bethesda, Maryland

**Keywords:** ABCD, Adolescent, Brain, Polygenic score, Risk factors, Substance use

## Abstract

**Background:**

Substance use disorders (SUDs) are moderately to highly heritable and develop in stages that typically begin in adolescence or young adulthood. The predominant use of cross-sectional adult samples to study SUDs and their genetic liability leaves it unclear whether their correlates may plausibly reflect predispositional risk and/or arise as a consequence of exposure.

**Methods:**

We conducted a phenome-wide association study (PheWAS) of polygenic scores (PGSs) for SUDs (i.e., problematic alcohol use, tobacco use disorder, cannabis use disorder [CUD], opioid use disorder [OUD], and a general addiction risk factor) in children ages 8 to 13 years (*n*_European ancestry_ = 5556; *n*_African American ancestry_ = 1584) enrolled in the Adolescent Brain Cognitive Development (ABCD) Study. Up to 1697 phenotypes were examined across several domains, including mental health, substance use, physical health, cognition, and screen media.

**Results:**

The PheWAS yielded 309 significant associations with the SUD PGS, particularly among mental health, sleep, screen time, and caffeine consumption phenotypes. Most associations remained significant in substance-naïve participants. The CUD PGS had the largest number of associations and was particularly associated with externalizing behavior in both genetic ancestry subsamples. OUD PGS associations were enriched for cognitive and neighborhood environmental phenotypes. Neuroimaging phenotypes (e.g., resting-state functional connectivity) partially accounted for associations between the SUD PGS and screen time phenotypes.

**Conclusions:**

Genetic liability to SUDs is expressed during childhood as potentially modifiable risk factors that could be targeted to attenuate its expression in adolescence and adulthood.

## Plain Language Summary

Genetic liability to substance use disorders is expressed in youth before substance use onset. Among up to 5556 children ages 8 to 13 years, a higher genetic predisposition to substance use disorders was linked to mental health symptoms, sleep issues, and higher screen time and media use. Identifying these early, potentially modifiable indicators offers clear targets for future research and the development of early interventions.

Substance use disorders (SUDs) are common and heritable (50%–60%) 1, 2 conditions that significantly and adversely impact individuals, families, and society 3, 4, 5, underscoring the importance of leveraging etiologic understanding to uncover ways to reduce risk. While typical onset of SUDs occurs during late adolescence and early adulthood (6), a host of behavioral factors have been implicated during adolescence as correlates of subsequent SUD, including early initiation of substance use, externalizing behaviors such as inattentiveness and impulsivity, and low mood 7, 8, 9. Psychosocial factors, such as family conflict, engagement with substance-using peers, resource scarcity, and neighborhood adversity, alongside the prominent role of family history of substance misuse and other mental health conditions, have also been linked to later substance use (10). A related literature has outlined several sequelae of SUDs, including socioeconomic and interpersonal difficulties, a heightened likelihood of mood and anxiety disorders, and sleep disturbances 11, 12, 13. Longitudinal studies have shaped our understanding of SUD precursors and sequelae. However, many have been conducted in high-risk cohorts that may have limited generalizability or within cohorts with established substance use histories that hamper the ability to distinguish predispositional risk from effects of exposure. This manner of studying psychosocial factors in individuals with a history of substance use precludes the investigation of whether these same factors might have preceded the onset of substance use and relate to substance use via overlapping genetic and environmental pathways. For example, sleep disturbances are often evaluated as a consequence of SUD. Tracing whether sleep disturbances are evident in youth, even prior to the onset of substance use, would inform etiology and provide tangible entry points for prevention and intervention efforts that may directly or indirectly mitigate the likelihood of SUD development.

SUDs are familial, with genetic effects explaining 50% to 70% of variation 14, 15. A historical literature studying offspring of individuals with histories of SUDs established that, even prior to SUD onset, youth with family histories of SUD were more likely to exhibit externalizing and internalizing behaviors during adolescence (16). However, few of these studies were able to fully evaluate the range of behaviors and experiences that manifest during childhood and correlate with elevated familial risk. More recently, large and well-powered genome-wide association studies (GWASs) of SUDs have enabled the construction of polygenic scores (PGSs) to examine how genetic liability may manifest phenotypically across the lifespan. Instead of examining select, hypothesized risk and resiliency factors associated with genetic propensity to SUDs, phenome-wide association studies (PheWASs) offer the opportunity to search a broad range of behaviors and experiences in an agnostic, unbiased manner, thus serving both to support or refute hypotheses and identify credible new correlates of SUD liability that can guide future hypotheses. PheWASs of SUD PGSs (SUD_PGS__s_) in adults have identified broad pleiotropic associations across mental and physical health conditions 17, 18, and PheWASs of other phenotypes have led to novel causal hypotheses that can be further tested (19). As the first serious symptoms of SUDs typically arise in late adolescence and/or early adulthood (6), the PheWAS approach could be particularly useful in samples of youths. For example, findings could reflect early markers of genetic susceptibility to SUDs and highlight potential mechanisms through which predispositional risk manifests, prior to the typical onset of substance use. This approach can further contextualize causal interpretations of phenotypic associations between SUDs and other behaviors. Thus, just as GWASs have led to profound new insights into disease etiology through unbiased screening of common variation across the genome, PheWASs in youth have the potential to pinpoint new risk and resilience factors before the onset of SUDs.

Here, to identify correlates of genetic liability to SUDs in childhood, we conducted a PheWAS of PGSs for problematic alcohol use (PAU), tobacco use disorder (TUD), cannabis use disorder (CUD), opioid use disorder (OUD), and a general addiction risk factor (ADDrf) in individuals genetically similar to African and European reference populations in the Adolescent Brain Cognitive Development (ABCD) Study at ages 8 to 11 (baseline; *n* = 1584 and 5556, respectively) and 10 to 13 (follow-up; *n* = 1273 and 5048, respectively), largely prior to substance use onset. We tested associations between each PGS and up to 1697 psychosocial and health-related phenotypes and 421 imaging-derived phenotypes. Secondary analyses evaluated whether associations persisted among substance-naïve participants to rule out potential confounding by concurrent substance use. Furthermore, given that genetic risk for SUDs may be expressed as externalizing problems prior to the onset of substance use, we conducted additional analyses covarying for externalizing behavior. Ultimately, phenotypes identified as correlates of genetic liability to SUDs during childhood prior to SUD expression, and in many cases substance exposures, may reflect putative risk factors through which genetic liability is expressed. These phenotypes could serve as accessible, malleable targets for family-based education/intervention to improve the health of youths at genetic risk for SUDs.

## Methods and Materials

### Participants

The ABCD Study is a longitudinal study of adolescent development in the United States (20). Our analyses were restricted to individuals whose genomic ancestry resembled African (*n* = 1584; mean ± SD age = 9.90 ± 0.61 years, 50.0% female) and European (*n* = 5556, 9.93 ± 0.63 years, 47.0% female) reference populations (hereafter referred to as African [AFR]- and European [EUR]-like genetic ancestry, respectively) (Table 1). Due to the lack of well-powered GWASs in other ancestral populations and challenges with cross-ancestry portability of summary data 21, 22, we did not analyze data from other genetic ancestries (*n* = 3959). A transancestral meta-analysis of the AFR and EUR associations is presented in the Supplement. Parental/caregiver written and child verbal informed consent/assent were given for a protocol approved by a centralized institutional review board and those at each data collection site (*n* = 21) (https://abcdstudy.org/sites/abcd-sites.html).

**Table 1:** Sample Descriptive Statistics

Variable	African-Like Ancestry, *n* = 1584	European-Like Ancestry, *n* = 5556
Sex, Female	792 (50.0%)	2612 (47.0%)
Age, Years	9.90 (0.60)	9.93 (0.63)
Household Income		
<$35,000	725 (54.2%)	375 (7.1%)
$35,000–$49,999	176 (13.2%)	283 (5.3%)
$50,000–$74,999	192 (14.3%)	717 (13.5%)
$75,000–$99,999	97 (7.2%)	896 (16.9%)
$100,000–$199,999	124 (9.3%)	2178 (41.1%)
≥$200,000	24 (1.8%)	849 (16.0%)
Highest Caregiver Education		
Less than high school	141 (8.9%)	25 (0.45%)
High school degree/equivalent	400 (25.3%)	188 (3.4%)
Some college/associate degree	621 (39.3%)	1046 (18.8%)
College degree	214 (13.6%)	1753 (31.6%)
Master’s degree	167 (10.6%)	1723 (31.0%)
Doctorate/professional degree	36 (2.3%)	820 (14.8%)

Values are presented as mean (SD) or *n* (%). Sample descriptive information is from the baseline wave of the Adolescent Brain Cognitive Development Study.

### Nonimaging Phenotypes

At the baseline and follow-up time points, 6194 and 10,156 nonimaging phenotypes were considered, respectively. Following exclusion of irrelevant (e.g., administrative questions, number of items missed for a specific scale) and redundant (e.g., from measures with multiple scoring methods, such as the NIH Toolbox) phenotypes and phenotypes with insufficient nonmissing data (i.e., ≤100 data points or ≤100 endorsements of minority categories of categorical variables), there were 940 and 1271 baseline phenotypes and 1357 and 1697 follow-up phenotypes for AFR and EUR individuals, respectively (Tables S1–S8).

Phenotypes were grouped into domains for descriptive and interpretative purposes: 1) identity (*n*_phenotypes_ = 0–40 across waves), 2) cognition (*n*_phenotypes_ = 8–13), 3) physical development (*n*_phenotypes_ = 18–31), 4) physical health (*n*_phenotypes_ = 39–84), 5) prenatal/postnatal and early development (*n*_phenotypes_ = 40–60 [baseline only]), 6) life events (*n*_phenotypes_ = 2–162), 7) family environment (*n*_phenotypes_ = 64–111), 8) neighborhood/external environment (*n*_phenotypes_ = 48–123), 9) peer environment (*n*_phenotypes_ = 10–38), 10) school related (*n*_phenotypes_ = 29–45), 11) screen time/media use (*n*_phenotypes_ = 18–106), 12) substance use and perceptions (*n*_phenotypes_ = 19–75), 13) substance-related environment (*n*_phenotypes_ = 12–52), 14) family behavior and mental health (*n*_phenotypes_ = 137–239), and 15) child behavior and mental health (*n*_phenotypes_ = 464–663) (Tables S1–S8).

### Imaging Phenotypes

Imaging phenotypes included cortical and subcortical volume, thickness, and surface area (*n*_phenotypes_ = 254); resting-state functional connectivity (*n*_phenotypes_ = 91); and white matter fractional anisotropy (FA) and mean diffusivity (MD) (*n*_phenotypes_ = 76). A detailed description of the imaging acquisition procedures and processing and analysis methodology in the ABCD Study can be found elsewhere 23, 24. Following quality control based on ABCD guidelines, 1448 to 1543 AFR and 5294 to 5517 EUR individuals were included in analyses of these phenotypes 23, 24.

### Polygenic Scores

See the Supplement for information on genotyping, quality control, and imputation. PGSs were computed using PRS-CS [EUR (25)] and PRS-CSx [AFR (26)]. Parameters for both PRS-CS and PRS-CSx included default phi parameters (*n*_iter = 10,000; *n*_burnin = 5000). PRS-CS used the auto function, and in PRS-CSx, the meta flag was set to TRUE. The PLINK version 2.0 (27) --score command was used to calculate PGSs for each individual by summing all variants weighted by the inferred posterior effect size.

The following SUD GWAS summary statistics were used: PAU [EUR *n* = 903,147 (3)], alcohol use disorder (AUD) [AFR *n* = 122,571, supplemented with *n* = 753,248 EUR (3)], TUD [AFR *n* = 114,420; EUR *n* = 739,895 (28)], CUD [AFR *n* = 123,208; EUR *n* = 886,025 (29)], OUD [AFR *n* = 84,877; EUR *n* = 554,186 (30)], and the ADDrf [AFR *n* = 92,630; EUR *n* = 1,025,550 (31)] (Table 2).

**Table 2:** Discovery GWAS Used to Generate PGSs

GWAS Phenotype	Discovery GWAS	Ancestry and *n*
AUD/PAU^a^	(3)	AUD: African *n* = 122,571 supplemented with European *n* = 739,895 PAU: European *n* = 903,147
TUD	(28)	African *n* = 114,420^b^ European *n* = 739,895
CUD	(29)	African *n* = 123,208^b^; European *n* = 886,025
OUD	(30)	African *n* = 84,877^b^; European *n* = 554,186
ADDrf	(31)	African *n* = 92,630^b^; European *n* = 1,025,550

ADDrf, addiction risk factor; AUD, alcohol use disorder; CUD, cannabis use disorder; GWAS, genome-wide association study; OUD, opioid use disorder; PAU, problematic alcohol use; PGS, polygenic score; TUD, tobacco use disorder.

a The cross-ancestry genetic-effect correlation between PAU and AUD is 0.71 (3).

b African-like ancestry samples were supplemented with European-like ancestry samples in PRS-CSx.

### Nonimaging Analyses

Primary analyses examined associations between each PGS and all curated phenotypes, separately for each ancestry group and time point. Continuous variables with skew ≥ |1.96| were winsorized to 3 SDs and, if skew remained ≥|1.96|, inverse normal transformed; all other continuous variables were *z* scored. Linear (for continuous outcomes) and generalized linear (for dichotomous outcomes) mixed-effects models were fit to the data using the lme4 package in R (32), with random intercepts for family and data collection site and the following fixed-effects covariates: the first 10 within-ancestry principal components, age (in months) at assessment, biological sex at birth (female and male), and visit type (in person, hybrid, or remote indicator for assessments completed during the COVID-19 pandemic). Models that did not converge due to insufficient variability in covariates or random effects were modified to exclude problematic parameters (e.g., biological sex assigned at birth was removed from pubertal development models, where phenotypes were assessed separately for boys and girls).

### Statistical Significance

Statistical significance was determined via Bonferroni correction for the number of tests (i.e., 940 and 1271 tests at baseline, 1361 and 1697 tests at follow-up), separately for each PGS. False discovery rate (FDR)–corrected *p* values are also presented in the Supplement, as the Bonferroni approach may be overly stringent, given the correlations among phenotypes.

### Enrichment Analyses

To identify domains enriched for associations with each PGS, we conducted 1-sided hypergeometric tests using the phyper() function in R, inputting the total number of phenotypes, Bonferroni-corrected significant associations, category size, and category-level significant associations for each PGS (33). This approach yields a *p* value and enrichment ratio (observed hits/expected hits) for each PGS-domain pair, indicating whether there were more associations than expected by chance. Benjamini-Hochberg FDR correction was applied across the resulting 70 to 75 *p* values (5 PGSs × 14–15 domains).

### Imaging Analyses

Linear mixed-effects models tested the associations between each of the PGSs and imaging metrics, which were also standardized and scaled. In addition to the covariates noted above, the following were included: magnetic resonance imaging (MRI) scanner type (Siemens, Philips, or GE), motion (motion correction for resting-state functional connectivity [RSFC] and diffusion-weighted imaging [DWI] analyses), and global metrics (i.e., total cortical volume and surface area, mean thickness, total subcortical volume, mean FA, and MD for analyses with regional metrics as outcomes).

### Statistical Significance

Bonferroni correction was applied to 1) all *p* values (*n* = 421) and 2) separately to all *p* values within each modality (i.e., global, cortical volume, surface area, thickness, subcortical volume, resting-state functional MRI, DWI FA, DWI MD) within each PGS model, with FDR correction also being reported in the Supplement.

Imaging analyses were repeated in the substance-naïve subsamples.

### Secondary Analyses

As detailed in the Supplement, secondary analyses were conducted as follows: 1) within substance-naïve samples (i.e., individuals who had not initiated substance use at the point of assessment), 2) with the 4 specific SUD_PGS__s_ entered simultaneously into a model, 3) with phenotypes pruned based on their correlation, 4) PGS × time interactions, 5) PGS × sex interactions, 6) within-family PGS, 7) inclusion of reported externalizing behavior as a covariate, 8) mediation models estimating whether neuroimaging-derived phenotypes indirectly link PGSs and behavioral associations, and 9) transancestry meta-analysis.

## Results

### Nonimaging: EUR-Like Genetic Ancestry

#### Broad Patterns

Each SUD_PGS_ (PAU, TUD, CUD, OUD, and ADDrf) (Table 2) was associated with 10 to 71 baseline (0.79%–5.59% of total phenotypes) and 19 to 136 follow-up (1.12%–8.01%) phenotypes following Bonferroni correction (*p*_baseline_ < 3.93 × 10^−5^, *p*_follow-up_ < 2.95 × 10^−5^), with their average absolute standardized betas and odds ratios ranging from 0.049 to 0.074 (mean = 0.061) and 1.25 to 1.40 (mean = 1.32), respectively (Figures 1 and 2; Tables S9 and S10; Figures S1 and S2). Across time points but varying by PGS, the screen time/media use, cognition, substance use and perceptions, and school-related domains had the greatest percentage of significantly associated phenotypes (up to 50%) (Table 3 and Figure 2; Tables S9 and S10).

**Figure 1:**
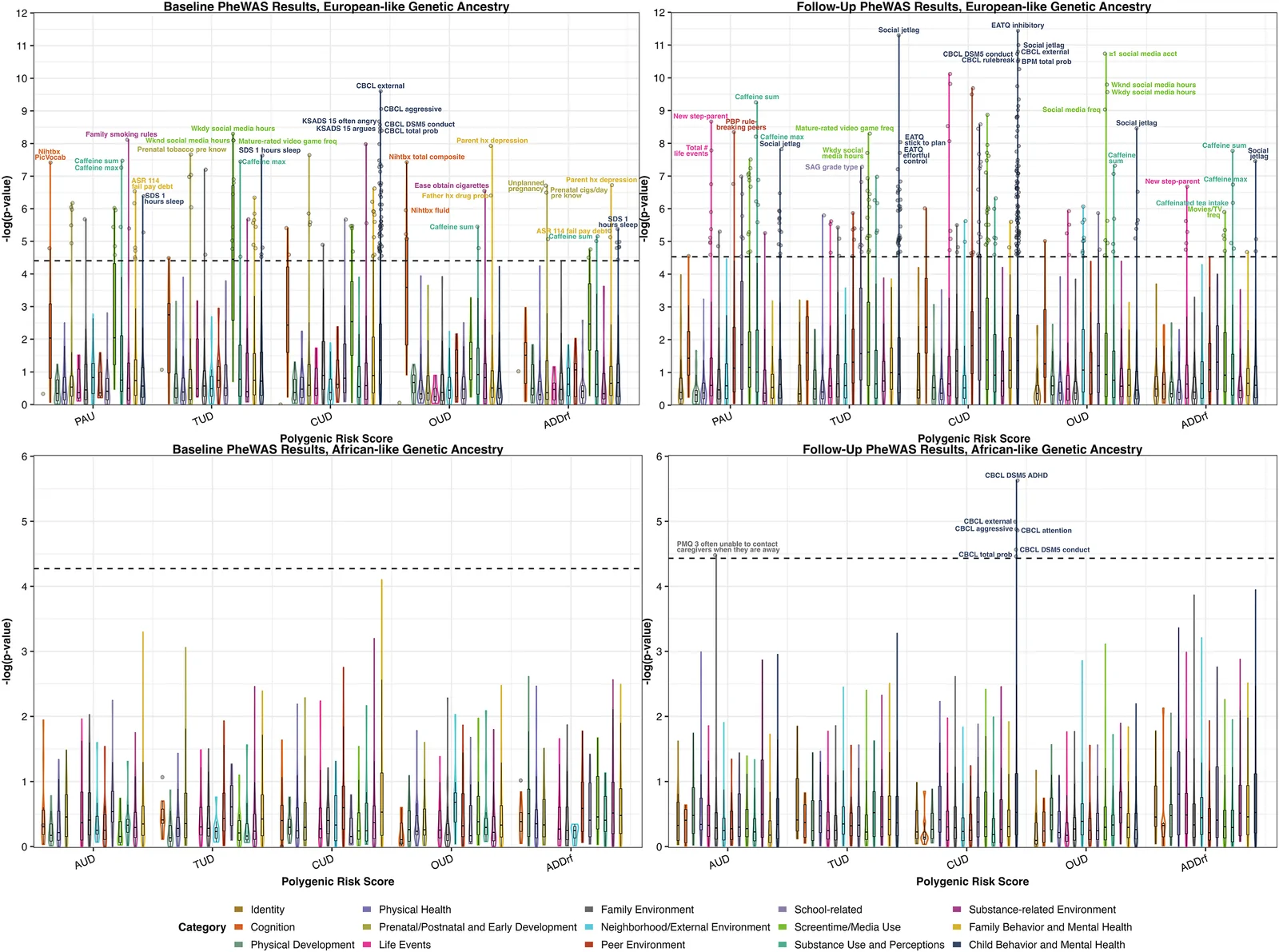
Manhattan and violin plots depicting the number, distribution, and types of phenotypes, within each category, associated with each of the 5 examined PGSs. The x-axes are the 5 PGSs, the y-axes are the negative log of the *p* value of association (higher value indicates stronger association), and color indicates phenotypic category. The dashed horizontal lines denote the Bonferroni-significant threshold (i.e., .05/number of phenotypes). The violin plots represent the distribution of −log(*p* values) within each category, separately for each PGS. Boxplots within each violin plot show the median −log(*p* value of association), and the boxes encompass the middle 50% of associations. ADDrf, addiction risk factor; ADHD, attention-deficit/hyperactivity disorder; ASR, Adult Self-Report; AUD, alcohol use disorder; BPM, Brief Problem Monitor; CBCL, Child Behavior Checklist; CUD, cannabis use disorder; EATQ, Early Adolescent Temperament Questionnaire; KSADS, Kiddie Schedule for Affective Disorders and Schizophrenia; OUD, opioid use disorder; PAU, problematic alcohol use; PBP, Peer Behavior Profile; PGS, polygenic score; PheWAS, phenome-wide association study; PMQ, Parental Monitoring Questionnaire; SAG, School Attendance and Grades; SDS, Sleep Disturbance Scale; TUD, tobacco use disorder.

**Figure 2:**
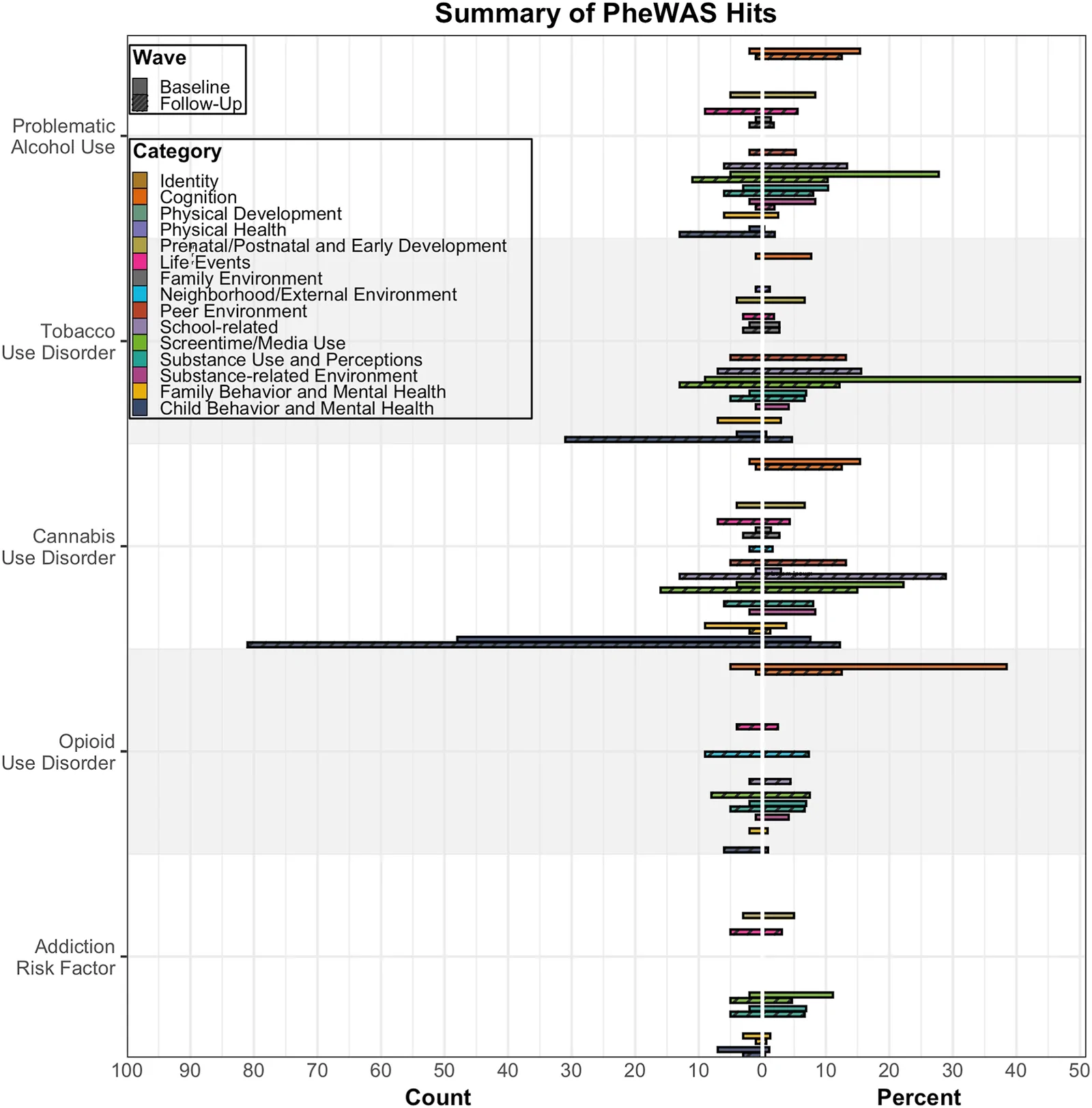
Summary of the number (count, left) and percentage (right) of associations observed between each PGS (y-axis) and phenotypes in each category, for European-like genetic ancestry. Colors denote different categories, and solid and dashed bars represent baseline and 2-year follow-up associations, respectively. Percent reflects the percentage of phenotypes in a category associated with a particular PGS of the total number of phenotypes in that category. PGS, polygenic score; PheWAS, phenome-wide association study.

**Table 3:** Summary of Associations by Phenotype Domain

	Total	Problematic Alcohol Use PGS		Tobacco Use Disorder PGS		Cannabis Use Disorder PGS		Opioid Use Disorder PGS		Addiction Risk Factor PGS	
		FDR	Bonf	FDR	Bonf	FDR	Bonf	FDR	Bonf	FDR	Bonf
Baseline											
Child Behavior and MH	634	37 (5.8%)	2 (0.32%)	90 (14.2%)	4 (0.63%)	239 (37.7%)	48 (7.6%)	23 (3.6%)	0 (0.0%)	38 (6.0%)	7 (1.1%)
Family Behavior and MH	239	20 (8.4%)	6 (2.5%)	40 (16.7%)	7 (2.9%)	70 (29.3%)	9 (3.8%)	12 (5.0%)	2 (0.84%)	12 (5.0%)	3 (1.3%)
Substance-Related Environment	24	4 (16.7%)	2 (8.3%)	4 (16.7%)	1 (4.2%)	5 (20.8%)	2 (8.3%)	3 (12.5%)	1 (4.2%)	4 (16.7%)	0 (0.0%)
Substance Use and Perceptions	29	5 (17.2%)	3 (10.3%)	7 (24.1%)	2 (6.9%)	4 (13.8%)	0 (0.0%)	4 (13.8%)	2 (6.9%)	6 (20.7%)	2 (6.9%)
Screentime and Media Use	18	8 (44.4%)	5 (27.8%)	15 (83.3%)	9 (50.0%)	11 (61.1%)	4 (22.2%)	2 (11.1%)	0 (0.0%)	9 (50.0%)	2 (11.1%)
School Related	34	1 (2.9%)	0 (0.0%)	6 (17.6%)	0 (0.0%)	9 (26.5%)	1 (2.9%)	0 (0.0%)	0 (0.0%)	1 (2.9%)	0 (0.0%)
Peer Environment	11	0 (0.0%)	0 (0.0%)	1 (9.1%)	0 (0.0%)	2 (18.2%)	0 (0.0%)	0 (0.0%)	0 (0.0%)	0 (0.0%)	0 (0.0%)
Neighborhood/External Environment	48	2 (4.2%)	0 (0.0%)	3 (6.3%)	0 (0.0%)	1 (2.1%)	0 (0.0%)	0 (0.0%)	0 (0.0%)	0 (0.0%)	0 (0.0%)
Family Environment	74	3 (4.1%)	1 (1.4%)	8 (10.8%)	2 (2.7%)	19 (25.7%)	1 (1.4%)	4 (5.4%)	0 (0.0%)	5 (6.8%)	0 (0.0%)
Life Events	6	0 (0.0%)	0 (0.0%)	2 (33.3%)	0 (0.0%)	0 (0.0%)	0 (0.0%)	0 (0.0%)	0 (0.0%)	0 (0.0%)	0 (0.0%)
Prenatal/Postnatal and Early Development	60	7 (11.7%)	5 (8.3%)	7 (11.7%)	4 (6.7%)	9 (15.0%)	4 (6.7%)	4 (6.7%)	0 (0.0%)	7 (11.7%)	3 (5.0%)
Physical Health	61	1 (1.6%)	0 (0.0%)	4 (6.6%)	0 (0.0%)	4 (6.6%)	0 (0.0%)	2 (3.3%)	0 (0.0%)	2 (3.3%)	0 (0.0%)
Physical Development	19	0 (0.0%)	0 (0.0%)	2 (10.5%)	0 (0.0%)	0 (0.0%)	0 (0.0%)	0 (0.0%)	0 (0.0%)	0 (0.0%)	0 (0.0%)
Cognition	13	4 (30.8%)	2 (15.4%)	8 (61.5%)	1 (7.7%)	9 (69.2%)	2 (15.4%)	8 (61.5%)	5 (38.5%)	2 (15.4%)	0 (0.0%)
Identity	1	0 (0.0%)	0 (0.0%)	0 (0.0%)	0 (0.0%)	0 (0.0%)	0 (0.0%)	0 (0.0%)	0 (0.0%)	0 (0.0%)	0 (0.0%)

Two-Year Follow-Up											
Child Behavior and MH	663	104 (15.7%)	13 (2.0%)	172 (25.9%)	31 (4.7%)	261 (39.4%)	81 (12.2%)	42 (6.3%)	6 (0.9%)	48 (7.2%)	3 (0.5%)
Family Behavior and MH	158	9 (5.7%)	0 (0.0%)	31 (19.6%)	0 (0.0%)	45 (28.5%)	2 (1.3%)	6 (3.8%)	0 (0.0%)	6 (3.8%)	1 (0.63%)
Substance-Related Environment	52	6 (11.5%)	1 (1.9%)	7 (13.5%)	0 (0.0%)	9 (17.3%)	0 (0.0%)	4 (7.7%)	0 (0.0)	3 (5.8%)	0 (0.0%)
Substance Use and Perceptions	75	21 (28.0%)	6 (8.0%)	17 (22.7%)	5 (6.7%)	24 (32.0%)	6 (8.8%)	13 (17.3%)	5 (6.7%)	11 (14.7%)	5 (6.7%)
Screentime and Media Use	107	30 (28.0%)	11 (10.3%)	51 (47.7%)	13 (12.1%)	44 (41.1%)	16 (15.0%)	26 (24.3%)	8 (7.5%)	25 (23.4%)	5 (4.7%)
School Related	45	19 (42.2%)	6 (13.3%)	19 (42.2%)	7 (15.6%)	27 (60.0%)	13 (28.9%)	14 (31.1%)	2 (4.4%)	12 (26.75%)	0 (0.0%)
Peer Environment	38	12 (31.6%)	2 (5.3%)	14 (36.8%)	5 (13.2%)	19 (50.0%)	5 (13.2%)	7 (18.4%)	0 (0.0%)	7 (18.4%)	0 (0.0%)
Neighborhood/External Environment	123	7 (5.7%)	0 (0.0%)	15 (12.2%)	0 (0.0%)	11 (8.9%)	2 (1.6%)	31 (25.2%)	9 (7.3%)	8 (6.5%)	0 (0.0%)
Family Environment	111	9 (8.1%)	2 (1.8%)	18 (16.2%)	3 (2.7%)	23 (20.7%)	3 (2.7%)	7 (6.3%)	0 (0.0%)	6 (5.4%)	0 (0.0%)
Life Events	162	25 (15.4%)	9 (5.6%)	16 (9.9%)	3 (1.9%)	26 (16.0%)	7 (4.3%)	11 (6.8%)	4 (2.5%)	16 (9.9%)	5 (3.1%)
Physical Health	84	1 (1.2%)	0 (0.0%)	6 (7.1%)	1 (1.2%)	4 (4.8%)	0 (0.0%)	2 (2.4%)	0 (0.0%)	0 (0.0%)	0 (0.0%)
Physical Development	31	0 (0.0%)	0 (0.0%)	1 (3.2%)	0 (0.0%)	2 (6.5%)	0 (0.0%)	1 (3.2%)	0 (0.0%)	1 (3.2%)	0 (0.0%)
Cognition	8	2 (25.0%)	1 (12.5%)	4 (50.0%)	0 (0.0%)	5 (62.5%)	1 (12.5%)	3 (37.5%)	1 (12.5%)	1 (12.5%)	0 (0.0%)
Identity	40	4 (10.0%)	0 (0.0%)	9 (22.5%)	0 (0.0%)	1 (2.5%)	0 (0.0%)	2 (5.0%)	0 (0.0%)	2 (5.0%)	0 (0.0%)

Summary of the number of significant associations per domain, PGS, and assessment time point. Percentages in parentheses are the percent of significantly associated phenotypes of the total number of phenotypes assessed in that domain.

Bonf, Bonferroni; FDR, false discovery rate; MH, mental health; PGS, polygenic score.

#### Child Behavior and Mental Health

SUD_PGS__s_—especially the CUD_PGS_ and TUD_PGS_—exhibited multiple positive associations with mental health problems, particularly those related to externalizing behavior. Additional associations included greater psychotic-like experiences (PAU_PGS_, TUD_PGS_, and CUD_PGS_), youth- or teacher-reported total behavior problems (all except ADDrf_PGS_), and greater sleep problems (e.g., fewer hours, total sleep disturbances, and social jet lag [i.e., discrepancy between weekday and weekend sleep times]). The CUD_PGS_ was significantly enriched for associations within this domain at both waves (*p*_hyper_ ≤ 1.46 × 10^−3^, *p*_hyper_FDR_ ≤ .022) (Table 4). For the 390 phenotypes measured at both time points, 11% to 53% of Bonferroni-significant associations persisted, with follow-up generally showing more associations.

**Table 4:** Enrichment Results

PGS	*K*	Category	*n*	*k*	Expected	Enrichment Ratio	*p*	*p*_FDR_
Baseline								
PAU	26	Cognition	13	2	0.266	7.52	.027	.103
	26	Family environment	74	1	1.514	0.661	.793	.999
	26	Screentime/media use	18	5	0.368	13.6	1.71 × 10^−5^	2.57 × 10^−4^∗
	26	Child behavior and mental health	634	2	13.0	0.154	.999	.999
	26	Family behavior and mental health	239	6	4.89	1.23	.361	.903
	26	Prenatal/postnatal and early development	60	5	1.23	4.07	.006	.045∗
	26	Substance use and perceptions	29	3	0.593	5.06	.020	.098
	26	Substance-related environment	24	2	0.491	4.07	.084	.253
TUD	30	Cognition	13	1	0.307	3.26	.268	.985
	30	Family environment	74	2	1.75	1.15	.531	.999
	30	Screentime/media use	18	9	0.425	21.2	2.62 × 10^−11^	3.93 × 10^−10^∗
	30	Child behavior and mental health	634	4	15.0	0.267	.999	.999
	30	Family behavior and mental health	239	7	5.64	1.24	.328	.985
	30	Prenatal/postnatal and early development	60	4	1.42	2.82	.049	.368
	30	Substance use and perceptions	29	2	0.685	2.92	.148	.739
	30	Substance-related environment	24	1	0.566	1.77	.439	.999
CUD	71	Cognition	13	2	0.73	2.75	.161	.807
	71	Family environment	74	1	4.13	0.242	.988	.999
	71	School related	34	1	1.90	0.527	.862	.999
	71	Screentime/media use	18	4	1.01	3.98	.015	.113
	71	Child behavior and mental health	634	48	35.4	1.36	1.46 × 10^−3^	.022∗
	71	Family behavior and mental health	239	9	13.4	0.670	.941	.999
	71	Prenatal/postnatal and early development	60	4	3.35	1.19	.435	.999
	71	Substance-related environment	24	2	1.34	1.49	.392	.999
OUD	10	Cognition	13	5	0.102	48.9	1.15 × 10^−8^	1.73 × 10^−7^∗
	10	Family behavior and mental health	239	2	1.88	1.06	.588	.999
	10	Substance use and perceptions	29	2	0.228	8.77	.020	.152
	10	Substance-related environment	24	1	0.189	5.30	.174	.871
ADDrf	17	Screentime/media use	18	2	0.241	8.31	.023	.277
	17	Child behavior and mental health	634	7	8.48	0.825	.833	.999
	17	Family behavior and mental health	239	3	3.20	0.938	.647	.999
	17	Prenatal/postnatal and early development	60	3	0.803	3.74	.042	.277
	17	Substance use and perceptions	29	2	0.388	5.16	.055	.277
Follow-Up								
PAU	51	Cognition	8	1	0.240	4.16	.217	.608
	51	Life events	162	9	4.87	1.85	.048	.167
	51	Family environment	111	2	3.34	0.600	.859	.999
	51	Peer environment	38	2	1.14	1.75	.318	.741
	51	School related	45	6	1.35	4.44	1.81 × 10^−3^	.013∗
	51	Screentime/media use	107	11	3.22	3.42	2.11 × 10^−4^	2.96 × 10^−3^∗
	51	Substance use and perceptions	75	6	2.25	2.66	.022	.105
	51	Child behavior and mental health	663	13	19.9	0.652	.987	.999
	51	Substance-related environment	52	1	1.56	0.64	.800	.999
TUD	68	Physical health	84	1	3.37	0.297	.971	.999
	68	Life events	162	3	6.49	0.462	.966	.999
	68	Family environment	111	3	4.45	0.674	.836	.999
	68	Peer environment	38	5	1.52	3.28	.016	.075
	68	School related	45	7	1.80	3.88	1.64 × 10^−3^	.011∗
	68	Screentime/media use	107	13	4.29	3.03	2.01 × 10^−4^	2.82 × 10^−3^∗
	68	Substance use and perceptions	75	5	3.01	1.66	.178	.499
	68	Child behavior and mental health	663	31	26.6	1.17	.159	.499
CUD	136	Cognition	8	1	0.641	1.56	.488	.999
	136	Life events	162	7	13.0	0.539	.982	.999
	136	Family environment	111	3	8.90	0.337	.996	.999
	136	Neighborhood/external environment	123	2	9.86	0.203	.999	.999
	136	Peer environment	38	5	3.05	1.64	.183	.642
	136	School related	45	13	3.61	3.61	2.50 × 10^−5^	1.75 × 10^−4^∗
	136	Screentime/media use	107	16	8.58	1.87	8.98 × 10^−3^	.042∗
	136	Substance use and perceptions	75	6	6.01	0.998	.566	.999
	136	Child behavior and mental health	663	81	53.1	1.52	3.82 × 10^−7^	5.34 × 10^−6^∗
	136	Family behavior and mental health	158	2	12.7	0.158	.999	.999
OUD	35	Cognition	8	1	0.165	6.06	.154	.539
	35	Life events	162	4	3.34	1.20	.433	.999
	35	Neighborhood/external environment	123	9	2.54	3.55	5.80 × 10^−4^	7.58 × 10^−3^∗
	35	School related	45	2	0.928	2.16	.237	.664
	35	Screentime/media use	107	8	2.21	3.63	1.08 × 10^−3^	7.58 × 10^−3^∗
	35	Substance use and perceptions	75	5	1.55	3.23	.017	.079
	35	Child behavior and mental health	663	6	13.7	0.439	.999	.999
ADDrf	19	Life events	162	5	1.81	2.76	.029	.134
	19	Screentime/media use	107	5	1.20	4.17	5.18 × 10^−3^	.036∗
	19	Substance use and perceptions	75	5	0.84	5.95	1.06 × 10^−3^	.015∗
	19	Child behavior and mental health	663	3	7.42	0.404	.993	.999
	19	Family behavior and mental health	158	1	1.77	0.565	.845	.999

Only PGS-category pairs for which there was at least 1 Bonferroni-significant association are shown. *K* indicates the number of Bonferroni-significant associations for the PGS; *n* indicates the number of phenotypes in the category; *k* indicates the number of Bonferroni-significant associations for the PGS in that category. Expected = (*n*/1271) × *K* [baseline]; expected = (*n*/1697) × *K* [follow-up]; enrichment ratio = *k*/expected. *p* is the 1-sided *p* value, and *p*_FDR_ is provided for all 75 (baseline) or 70 (follow-up) tests.

ADDrf, addiction risk factor; CUD, cannabis use disorder; FDR, false discovery rate; OUD, opioid use disorder; PAU, problematic alcohol use; PGS, polygenic score; TUD, tobacco use disorder.

∗ *p*_FDR_ < .05.

#### Screen Time/Media Use

Each SUD_PGS_ was positively associated with frequency and time spent using social media. All SUD_PGS__s_ except the OUD_PGS_ were associated with weekend screen time and R-rated movie frequency, with additional SUD-specific associations observed (e.g., higher frequency of playing mature video games, weekend texting, and weekday TV/movie time). Both TUD_PGS_ and PAU_PGS_ were significantly enriched for associations within this domain at both waves (*p*_hyper_ ≤ 2.11 × 10^−4^, *p*_hyper_FDR_ ≤ 2.96 × 10^−3^) (Table 4). OUD_PGS_, ADDrf_PGS_, and CUD_PGS_ also exhibited significant enrichment at follow-up (*p*_hyper_ ≤ 8.98 × 10^−3^, *p*_hyper_FDR_ ≤ .042) (Table 4). Of 18 screen time/media phenotypes measured twice, 0% to 80% of those linked to an SUD_PGS_ at one time point replicated at the other.

#### Cognition

The OUD_PGS_ was negatively associated with more phenotypes related to cognitive performance (e.g., NIH Toolbox total composite, fluid intelligence) at baseline (*n* = 5) than any other PGS (*n* ≤ 2) and exhibited significant enrichment (*p*_hyper_ = 1.15 × 10^−8^, *p*_hyper_FDR_ = 1.73 × 10^−7^) (Table 4). Cognition phenotypes represented 50% of the significant baseline associations with the OUD_PGS_.

#### Substance Use and Perceptions

All SUD_PGS__s_ were associated with caffeine intake (e.g., maximum intake, summary score, and/or energy drink use). Associations also emerged for less perceived harm for regular smokeless tobacco use (all PGSs) and more positive cannabis use expectancies (CUD_PGS_). The ADDrf_PGS_ was significantly enriched for associations within this domain at follow-up (*p*_hyper_ = 1.06 × 10^−3^, *p*_hyper_FDR_ = .015). Of the 28 phenotypes in this domain, 0% to 75% of those with significant SUD_PGS_ links at one point remained significant at the other.

#### School Related

Only the CUD_PGS_ was associated with lower school performance at baseline, and all PGSs except the ADDrf_PGS_ were linked to lower school performance, increased likelihood of detention, and/or school disengagement at follow-up. At follow-up, CUD_PGS_, TUD_PGS_, and PAU_PGS_ showed significant enrichment for phenotypes in this domain (*p*_hyper_ ≤ 1.81 × 10^−3^, *p*_hyper_FDR_ ≤ .013) (Table 4).

#### Prenatal/Postnatal and Early Development

All SUD_PGS__s_, except the OUD_PGS_, were associated with unplanned pregnancy and prenatal tobacco exposure; PAU_PGS_ and CUD_PGS_ were also associated with prenatal cannabis exposure. The PAU_PGS_ showed significant enrichment for associations within this domain (*p*_hyper_ = 6.06 × 10^−3^, *p*_hyper_FDR_ = .046).

#### Family Behavior and Mental Health

All SUD_PGS__s_ were positively associated with a paternal history of drug problems and a parental history of depression. Additional positive associations included current caregiver externalizing-related and somatic problems (TUD_PGS_ and CUD_PGS_), with more associations being present at baseline.

#### Substance-Related Environment

PAU_PGS_, TUD_PGS_, CUD_PGS_, and OUD_PGS_ were associated with the presence of differential cigarette use rules among family members, greater cigarette accessibility, and/or peer tobacco use. Of the 22 phenotypes assessed at both waves, only baseline associations exhibited significant associations.

#### Neighborhood/External Environment

At follow-up, the CUD_PGS_ was positively associated with the experience of sexual orientation– and weight-related discrimination; the OUD_PGS_ was positively associated with several neighborhood adversity metrics (e.g., lower high-skill employment and child opportunity; higher number of individuals with a disability). The OUD_PGS_ demonstrated significant enrichment for associations within this domain (*p*_hyper_ = 5.80 × 10^−4^, *p*_hyper_FDR_ = 7.58 × 10^−3^) (Table 4).

#### Life Events

All PGSs were positively associated with stressful life event exposure measured at follow-up; all PGSs except the OUD_PGS_ were also associated with greater impact of these events.

#### Family Environment

SUD_PGS__s_ were associated with lower parent/caregiver educational attainment (TUD_PGS_, CUD_PGS_ at baseline), a parent/caregiver partner not being a biological parent (PAU_PGS_, TUD_PGS_ at both waves), caregiver-child conflict (TUD_PGS_, CUD_PGS_ at follow-up), and less frequent family dinners (PAU_PGS_ at baseline).

#### Peer Environment

At the follow-up wave, PAU_PGS_, TUD_PGS_, and CUD_PGS_ were positively associated with involvement with peers who break rules and have been suspended; TUD_PGS_ and CUD_PGS_ were associated with experiencing peer victimization.

#### Physical Health

The TUD_PGS_ was negatively associated with the number of extracurricular (largely sports) activities at follow-up.

#### Physical Development and Identity

No significant associations were observed with any phenotypes within the physical development or identity domains.

### Secondary Analyses: EUR-Like Genetic Ancestry

#### Substance Naïve

A majority (76.9%–100% across PGS and waves) of Bonferroni-significant associations in the total sample were Bonferroni significant in the substance-naïve subsample (Tables S11 and S12).

#### SUD_PGS_: Specificity

When all PGSs were entered simultaneously, the CUD_PGS_ (20–21 phenotypes predominantly related to externalizing), OUD_PGS_ (0–2 phenotypes related to neighborhood adversity, social media use), and TUD_PGS_ (2–3 phenotypes related to screen time/media use, mental health/behavior)—but not the PAU_PGS_—exhibited unique associations (Tables S13 and S14). On average, the CUD_PGS_ had the largest effects (1.2–1.8 times greater than any other PGS), followed by the TUD_PGS_, PAU_PGS_, and OUD_PGS_.

#### Phenotypic Pruning

Pruning intercorrelated phenotypes resulted in a domain-nonspecific monotonic reduction of significant associations (range = 0%–66.7%) (Tables S9 and S10; Figure S3).

#### Phenotypic Change

No significant SUD_PGS_ × time interactions emerged for the 803 phenotypes measured at both waves (Table S15).

#### Sex Moderation

Four SUD_PGS_ × sex interactions were observed (Tables S16 and S17; Figure S4).

#### Within Family

Few phenotypes were associated with within-family deviations in PGSs following Bonferroni correction (i.e., PAU_PGS_: increased caffeinated soda consumption; TUD_PGS_: increased caregiver restlessness, greater mood instability; CUD_PGS_: increased externalizing behavior) (Tables S18 and S19; Figure S5).

#### Accounting for Baseline Externalizing Scores

The majority (58.2%–100% across PGSs) of follow-up phenotypes that were previously Bonferroni-significant remained associated with the SUD_PGS__s_ after covarying for baseline Child Behavior Checklist externalizing scores (Table S20). These notably included measures related to caffeine intake, screen time, and sleep disturbance.

### AFR-Like Genetic Ancestry

Seven follow-up (and no baseline) phenotypes—primarily externalizing-related behaviors—were associated with SUD_PGS__s_ (Figure 1C, D; Figures S6 and S7; Tables S21 and S22). These associations were significantly correlated with associations observed in the EUR-like ancestry subsample for AUD/PAU_PGS_, TUD_PGS_, CUD_PGS_, and ADDrf_PGS_ (*r* = 0.089–0.39, *p* ≤ 1.45 × 10^−4^). Significant associations remained in the substance-naïve subsample (Tables S23 and S24). Secondary analyses revealed 1) unique associations between the CUD_PGS_ and externalizing behaviors (Tables S25 and S26), 2) no significant SUD_PGS_ × time or SUD_PGS_ × sex interactions (Tables S27–S29), and 3) that all CUD_PGS_ associations remained significant in within-family analyses (Tables S30 and S31).

### Imaging Results

Results were only significant in the EUR subsample. SUD_PGS__s_ were linked to RSFC (greater sensorimotor hand and sensorimotor mouth network connectivity [PAU_PGS_, CUD_PGS_]; frontoparietal and sensorimotor hand networks [CUD_PGS_]) and DWI (lower FA in the right corticospinal/pyramidal [TUD_PGS_], superior corticostriate [TUD_PGS_], and left superior corticostriate–parietal cortex [TUD_PGS_] tracts) metrics. These associations remained in the substance-naïve subsample (Tables S32 and S33).

### Indirect Effects Results

All RSFC and DWI metrics significantly indirectly linked SUD_PGS__s_ to follow-up phenotypes (Figures S8–S10; Tables S34 and S35).

### Cross-Ancestry Meta-Analysis

Between 5.71% and 64.3% of Bonferroni-significant associations in the EUR subsample were similarly significant in the transancestry meta-analysis. Many associations that did not survive correction in the meta-analysis exhibited heterogeneous effects across ancestry groups (Tables S36 and S37).

## Discussion

Genetic liability to SUDs (i.e., PAU_PGS_, TUD_PGS_, CUD_PGS_, OUD_PGS_, and ADDrf_PGS_) was strongly and most frequently associated with elevated externalizing behavior in both AFR- and EUR-like individuals and notably with more distal phenotypes related to screen time/media use, caffeine intake, sleep problems, family history of substance use and depression, and prenatal factors (e.g., unplanned pregnancy, prenatal substance exposure) among EUR individuals in our PheWAS of 8- to 13-year-old children. There was no evidence that these associations differed significantly between ages 8 to 11 and 10 to 13. Most associations persisted among substance-naïve youths and thus are more likely to reflect early markers of genetic liability to SUD—operating through direct genetic effects, gene-environment correlation, and/or other indirect pathways—than causal consequences of substance use. Certain correlates, such as screen time/media, caffeine use, and sleep, should be investigated as potentially modifiable markers of SUD risk. If these correlates are shown to indirectly link genetic liability to future substance use, they could be studied in the context of preventive policy and intervention.

SUD_PGS__s_ were strongly associated with childhood externalizing behavior (e.g., conduct problems, aggression, inattention). The few significant associations in the AFR subsample almost entirely comprised externalizing and related (e.g., impulsivity, school performance) phenotypes, several of which also exhibited significant transancestry consistency. Thus, our results reinforce the well-established shared genetic architecture (34) and evidence of heterotypic continuity, wherein early externalizing behaviors (e.g., attention-deficit/hyperactivity disorder, oppositional defiant disorder symptoms) represent an earlier manifestation of a broad externalizing predisposition, which later includes SUDs (35).

Caffeine intake, screen time/media use, and sleep problems—particularly robustly associated with SUD_PGS__s_—have been previously genetically and/or phenotypically correlated with externalizing problems 19, 20, 21, 22, 23, 24. However, our externalizing-adjusted PheWAS revealed that SUD_PGS_ associations with caffeine intake, screen time, and sleep disturbance at follow-up were partially independent. Whether these robust residual associations are due to pleiotropy that is exclusive to SUD genetic liability, a predisposition toward emotion dysregulation distinct from externalizing, and/or other explanations is worth exploring.

Reflecting the ubiquity of gene-environment correlation, SUD_PGS__s_ were associated with prenatal factors (e.g., tobacco exposure, unplanned pregnancy), having rule-breaking peers, experiencing victimization from and aggressing upon peers, experiencing stressful life events, living in a disadvantaged neighborhood, and having increased ease of access to cigarettes. Particularly during childhood, genetic liability is intertwined with passive exposure to environments shaped by those genetic susceptibilities, exacerbating their dual impact (36). These early, risky, and genotype-related environments have been linked to later substance use (37) and variability in brain development (38) that may hinder the emergence of the cognitive restraint necessary to deter progression to SUD, making them ideal targets for public health interventions. Our within-family analyses further underscore the likelihood that many of the observed associations partially reflect indirect pathways from genotype to behavior via shaping of the childhood environment, consistent with evidence of the predominance of familial environment in the etiology of the initial stages of substance use (39).

SUD_PGS__s_ were robustly linked to sleep problems and less sleep. Beyond the well-established acute effects of psychoactive substances and withdrawal on sleep 40, 41, growing evidence highlights a shared genetic liability underlying both sleep disruptions and substance-related phenotypes (42), including traits such as evening chronotype (43), consistent with our social jet lag findings (44). Regardless of whether these findings reflect yet another aspect of behavioral dysregulation, are consequent to the antisomnolent effects of caffeine intake and protracted exposure to screens, and/or are attributable to loci with pleiotropic effects on SUDs and sleep, youth with genetic susceptibility to SUDs should be monitored for sleep difficulties and their resultant impact on health, as our results suggest.

Consistent with small, difficult-to-detect brain-behavior associations (45), few statistically significant associations between the SUD_PGS__s_ and imaging metrics emerged. However, there were two notable findings. First, both PAU_PGS_ and CUD_PGS_ were associated with increased RSFC between the sensorimotor hand and sensorimotor mouth networks, and the CUD_PGS_ was also linked to increased RSFC between the frontoparietal and sensorimotor hand networks. These neural metrics indirectly linked these SUD_PGS__s_ to screen time behavior. A recent meta-analysis found some evidence that increased sensorimotor cortex connectivity is not only associated with SUD diagnosis but also with less craving, better SUD treatment response, and reduced consumption among individuals with SUD (46), and another study found positive associations with the presence of gaming disorder (47), suggesting a potential shared pathway for multiple addictive behaviors. Second, the TUD_PGS_ was associated with reduced white matter integrity in the superior cortical-striatal, corticospinal/pyramidal, and cortical-striate-parietal tracts—tracts connecting regions frequently implicated in theoretical and clinical studies of addiction 48, 49, and these brain metrics partially mediated the association between the PGS and screen time behavior. As findings were evident in substance-naïve youths, they raise the intriguing possibility of preexisting brain variability associated with SUD genetic susceptibility (49).

Lastly, while there is compelling support for genetic commonality across SUDs, captured here by ADDrf, some SUD-specific distinctions are noteworthy. The CUD_PGS_ was associated with significantly more mental health phenotypes, which may be due to its high loading with other externalizing constructs 34, 50 or due to the nature of electronic health record diagnoses, which, for CUD, likely reflect complicating additional psychopathology (51). On the other hand, the OUD_PGS_ was associated with fewer phenotypes overall, possibly due to its smaller size, but was uniquely associated with lower global cognitive performance and greater environmental adversity, consistent with prior genetic studies (30).

There are some limitations to the study. First, despite our inclusion of data from individuals of AFR-like genetic ancestry, the sample sizes of these discovery GWASs and the target AFR-like ABCD sample were small. Summary statistics for other global ancestral groups were absent. Second, data from the Million Veteran Program, which over-represents older male veterans, are a major component of the SUD GWAS from which PGSs were derived, potentially influencing associations; however, these GWASs appear to genetically correlate with GWASs from other samples [e.g., *r*_g_ = 0.66–1.00 28, 29], and our analyses of sex differences did not yield significant results. Third, there was little endorsement of substance use beyond alcohol sipping (52), so we were unable to examine associations with substance use phenotypes beyond alcohol sipping and caffeine consumption. Fourth, we elected to use Bonferroni correction, which may be overly conservative, given substantial interphenotype correlations. Because of this potential for false negatives, FDR-corrected and uncorrected *p* values are also presented. Fifth, because the individual GWASs used to compute the SUD_PGS__s_ were larger than those used to generate the ADDrf, differences between ADDrf and individual SUD_PGS_ results should be interpreted cautiously.

### Conclusions

By identifying associations between SUD PGSs and conditions observable well before the onset of problematic (or any) substance use, the current PheWAS provides insights into childhood behaviors and environments that may be harbingers of future problems. The current analyses do not test causal hypotheses regarding the role of these childhood phenotypes on later SUD, and PGSs alone cannot provide complete prognostic insight into multifactorial phenotypes such as SUD. However, many of the associated phenotypes (e.g., caffeine consumption, screen time and social media use, sleep problems, and cigarette availability) could plausibly represent modifiable targets. Interventions for these targets could be explored as ways to broadly benefit youth health and likely even more so for youth with greater genetic susceptibility.

## Supplementary Material

Supplemental Methods, Results, and Figures S1–S10

Tables S1–S37

Key Resources Table
