# Supplementary material for: Youth Correlates of Genetic Liability to Substance Use Disorders
Source: Biol Psychiatry Glob Open Sci. 2026 Sep 28;7(1):100835. doi: 10.1016/j.bpsgos.2026.100835 (PMC13760950; doi:10.1016/j.bpsgos.2026.100835)
Supplement: Supplementary file 1 [file mmc1.pdf]

# SUPPLEMENTARY INFORMATION

# Youth Correlates of Genetic Liability to Substance Use Disorders

Paul *et al.*

**Contents**

Genotyping, Quality Control, and Imputation ........ 2
Secondary Analyses ........ 2-4
European-like genetic ancestry ........ 4-8
Substance-naive PheWAS ........ 4-5
Simultaneous SUD PGS PheWAS ........ 5
Phenotypic pruning ........ 5-7
PGS-by-time moderation ........ 7
Sex moderation ........ 7
Within-family PGS effects ........ 8
PheWAS adjusted for phenotypic externalizing behavior ........ 8
African-like genetic ancestry ........ 9-10
Substance-naive PheWAS ........ 9
Simultaneous SUD PGS PheWAS ........ 9
Phenotypic pruning ........ 9
PGS-by-time moderation ........ 9
Sex moderation ........ 9
Within-family PGS effects ........ 9
PheWAS adjusted for phenotypic externalizing behavior ........ 9-10
Indirect effects through neuroimaging-derived phenotypes ........ 10
Cross-ancestry meta-analysis ........ 10-16
References ........ 17
Supplemental Figures ........ 20

***Genotyping, Quality Control, and Imputation.*** Saliva samples were genotyped on the Smokescreen array(1) by the Rutgers University Cell and DNA Repository (now incorporated with other companies as Sampled; https://sampled.com/). The Rapid Imputation and COmputational PIpeLIne for Genome-Wide Association Studies (RICOPILI(2)) was used to perform quality control (QC) on the 11,099 individuals with available ABCD Study phase 3.0 genotypic data, using RICOPILI's default parameters (genotyping call rate >98%, inbreeding coefficient (F) < ±0.2, sex checks). The 10,585 individuals who passed these QC checks were matched to broad self-reported racial groups using the ABCD Study parent survey. Of the 6,787 parents/caregivers indicating that their child's race was only "white," 5,561 of those individuals did not endorse any Hispanic ethnicity/origin. Of the 1,675 parents/caregivers who indicated that their child's race was only "Black," 1,584 did not endorse any Hispanic ethnicity/origin.

Next, variants were filtered to exclude those with missingness >2% and HWE p-value <1e-06. Using data from unrelated individuals (pi-hat ≤ 0.2) and an LD pruned set of common (MAF>0.05) and non-palindromic SNPs (and excluding MHC and chromosome eight inversion region), principal components analysis (PCA) was performed in RICOPILI using EIGENSTRAT(3) to confirm the genetic ancestry of these individuals. This was done by merging the ABCD Study data with the 1000 Genomes reference panel, computing the mean and standard deviations for the 1000 Genomes ancestry populations for the top 6 PCs, and establishing that the previously identified group participants were genetically similar to the 1000 Genomes European and African reference panels by assessing whether they fell within 3SDs of the mean for the top 6 PCs within the respective 1000 Genomes reference population. After another round of QC on these subsamples, 5,556 individuals of European ancestry and 1,584 individuals of African ancestry were retained in the analyses. Each ancestry subset was then imputed to the TOPMed imputation reference panel(3). Imputation dosages were converted to best-guess hard-called genotypes, and only SNPs with Rsq > 0.8, MAF > 0.01, missingness < 0.1, and HWE p-values >1E-06 were kept for PGS analyses.

**Polygenic Scoring.** PRS-CS(4), PRS-CSx(5), and PLINK v2.0(6) were used to compute polygenic scores (PGS) for the substance use disorder phenotypes described below. PRS-CS is a Bayesian polygenic prediction method that applies a continuous shrinkage prior to SNP effect estimates and infers posterior SNP weights. PRS-CSx is an extension of PRS-CSx that integrates summary statistics and LD reference panels from multiple ancestral groups to enable more accurate polygenic prediction in populations for which sufficiently powered GWAS may not exist. Parameters for both PRS-CS and PRS-CSx included default phi parameters and n_iter = 10,000, n_burnin = 5,000; PRS-CS used the auto function, and in PRS-CSx, the meta flag was set to TRUE. We used PRS-CS for the EUR sample where the ancestry-matched GWAS are well-powered, and the Meta weights from PRS-CSx for the AFR sample where borrowing from the EUR GWAS improves prediction. The PLINK –score command was used to calculate PGS for each individual by summing all variants weighted by the inferred posterior effect size.

### *Secondary Analyses*

**1) Substance-naive PheWAS**. The data were restricted to individuals who had not yet initiated any substance use (i.e., "substance-naive") to rule out effects of exposure (baseline: $n_{AFR}$=1,411, $n_{EUR}$=4,289; follow-up: $n_{AFR}$=1,125, $n_{EUR}$=3,704). Substance-naive individuals had non-missing substance use data at the point of assessment (i.e., baseline, follow-up) and did not report any substance use (including during religious ceremonies)

at any point up to and including the current assessment. For more information, see(7). The substance-naive samples did not differ from the full samples by sex, age, household income, or caregiver sex ($p \geq 0.079$). The primary PheWAS and imaging analyses were repeated in the substance-naive samples. Bonferroni correction adjusted for the number of tests, with phenotypes no longer passing endorsement criteria in the substance-naive subsamples excluded from analyses.

**2) Simultaneous SUD PGS PheWAS.** The four non-ADDrf SUD PGS were entered into PheWAS analyses simultaneously to identify potentially independent SUD PGS associations.

**3) Phenotypic pruning.** Given interrelatedness among phenotypes, PheWAS-significant phenotypes were pruned based on their degree of overlap as determined by pseudo $R^2$(8). First, linear mixed-effects models were used to estimate bivariate pseudo $R^2$ values (lme4(9) and MuMIn(10) R packages) between each PheWAS-significant phenotype, with random intercepts for family and research site. Second, for each $R^2$ threshold examined (0.05, 0.1, 0.2, 0.3, 0.4, 0.5, 0.6, 0.7, 0.8, 0.9, 1.0), the phenotype with the higher (i.e., less significant) PheWAS p-value within each phenotype pair whose $R^2$ value was at or above the $R^2$ threshold was iteratively removed until no remaining $R^2$ values met that threshold.

**4) PGS-by-time moderation.** For each of the 802 and 619 phenotypes assessed at both baseline and follow-up in EUR and AFR, respectively, each PGS (separately) was interacted with time point (baseline and follow-up), controlling for sex, person-mean age, the first 10 ancestral PCs, and all predictor-by-covariate interactions, and including random intercepts for individual, family, and site.

**5) Sex moderation.** To test whether biological sex at birth moderated associations between each PGS and phenotype, PGS-by-sex interactions were examined for all phenotypes with sufficient data from both boys and girls. All predictor-by-covariate interactions (e.g., PGS-by-age, sex-by-age) were included to appropriately control for confounding(11). Simple slopes and Johnson-Neyman intervals were computed for all statistically significant interactions using the "interactions" R package(12).

**6) Within-family PGS effects.** Within-family analyses, wherein the PGS is decomposed into between- (family-mean PGS) and within-family (each individual's deviation from the family-mean PGS) variance, offer a way to exploit family-based designs to more closely approximate whether PGS associations may plausibly reflect causal genetic effects on outcomes rather than confounding due to population stratification, assortative mating, and/or passive gene-environment correlation (13–15). The sample was restricted to families with ≥2 siblings in the ABCD study (baseline: $n_{EUR}$=1809 [n=893 families]; follow-up: $n_{AFR}$=316 [n=156 families], $n_{EUR}$=1655 [n=827 families] baseline AFR excluded due to lack of significant baseline associations). Family-mean PGS and each sibling's deviation from that family-mean PGS were entered into mixed effects models. Only phenotypes that were previously identified as significantly associated with each PGS, following Bonferroni correction, and that passed quality control criteria within the smaller sibling subsamples were included.

**7) PheWAS adjusted by phenotypic externalizing behavior.** Substance use disorders are commonly categorized within the externalizing spectrum of psychiatric disorders, given their overlapping phenotypic and genetic variance(16–18). Given the possibility that genetic risk for SUDs may be expressed earlier as externalizing problems prior to the onset of substance use, we tested whether follow-up associations are independent of baseline externalizing behaviors by including the baseline CBCL externalizing scale as a covariate in analyses for phenotypes exhibiting Bonferroni-corrected significant associations with SUD PGS.

**8) Indirect effects through neuroimaging-derived phenotypes.** To explore potential neural mechanisms linking genetic liability to its phenotypic expression, we tested whether baseline imaging-derived phenotypes indirectly linked PGS to follow-up phenotype. First, associations between baseline imaging metrics and two-year follow-up phenotypes were tested. Only imaging metrics and phenotypes that were significantly associated with the same PGS were included (i.e., survived Bonferroni correction in the full sample and were nominally significant ($p$<0.05) in the substance-naive subsample). Second, two mixed effects models were run using the mediation R package(19): the first estimated the association between the PGS and brain structure metric (a path), controlling for baseline age, sex, MRI scanner type, the first 10 PCs, and site ID, with a random intercept for family; the second estimated the association between the brain structure metric and the follow-up outcome (b path), additionally controlling for the PGS, COVID visit type, and follow-up (instead of baseline) age. The indirect effect and standard error were computed via bootstrapping, with 10,000 simulations. Site ID was included as a fixed effect because the mediation package only allows for a single level of nesting.

**9) Cross-ancestry meta-analysis.** The smaller discovery GWAS and target sample size in AFR may reduce power. To derive estimates more generalizable across ancestry groups, we used the **metafor** R package(20) to implement random-effects meta-analyses, using the ancestry-stratified effect estimates and standard errors from the primary PheWAS. P-values were Bonferroni-corrected for the (smaller) number of phenotypes analyzed in AFR (940 at baseline; 1,357 at follow-up).

***European-like Genetic Ancestry***

**Substance-naive PheWAS**. Most (PAU: 96.2%; TUD: 100%; CUD: 88.6%; OUD: 100%; ADDrf: 93.8%) associations between baseline phenotypes and SUD PGS remained significant in the substance-naive sample, following Bonferroni correction for the number of phenotypes (n=10-70; $|B|\geq0.025$, OR≥1.15, $p\leq1.04\text{e-}03$, $p_{\text{Bonferroni}}\leq0.048$; **Supplemental Table 11**). Associations between PAU PGS and frequency of watching R-rated movies; between CUD PGS and frequency of watching R-rated movies, CBCL externalizing items and DSM-5 depression, past pressured speech, caregiver rulebreaking problems, and total sleep problems; and between ADDrf PGS and caregiver failure to pay debts did not survive correction for multiple tests ($|B|\leq0.053$, OR=1.28, $p\geq7.17\text{e-}04$, $p_{\text{Bonferroni}}\geq0.050$; **Supplemental Table 11**).

Similarly, the majority (PAU: 84.3%; TUD: 85.1%; CUD: 76.9%; OUD: 97.1%; ADDrf: 84.2%) of associations between follow-up phenotypes and SUD PGS survived Bonferroni correction for the number of phenotypes (n=19-134) in the substance-naive sample ($|B|\geq0.026$, OR≥1.16, $p\leq1.08\text{e-}03$, $p_{\text{Bonferroni}}\leq0.048$; **Supplemental Table 12**). Associations between PAU PGS and several life events items (e.g., family member arrested, negative impact summary score), youth self-reported total problems and number of psychotic-like experiences, NIH Toolbox picture vocabulary, using one's phone in the middle of the night, and the number of friends who drink alcohol); TUD PGS and several child behavior and mental health phenotypes (i.e., self-reported hot temper, caregiver-reported destroying one's own things, suddenly not trusting others, volatile mood, "mania" summary score, and total sleep problems) and fewer sports and activities in the past year; CUD PGS and a number of youth- and caregiver-reported child behavior and mental health phenotypes (e.g., impulsive, aggressive, and internalizing behaviors and traits; bedtime reluctance), caregiver-child conflict, life events (losing close friend, family member

with an SUD), lower school involvement (excused absences, disliking school), screentime and media use (weekend time spent editing photos or videos to post on social media, weekday time spent texting, frustration when unable to use screen media), and positive cannabis expectancies (i.e., cannabis helps a person relax); OUD PGS and coffee intake; and ADDrf PGS and coffee intake, having an internet-connected device or TV in one's bedroom, and frequency of spending time on social media) did not survive correction for multiple tests ($|B| \leq 0.085$, OR≤1.25, $p \geq 3.91e-04$, $p_{Bonferroni} \geq 0.051$; **Supplemental Table 12**).

**Simultaneous SUD PGS PheWAS.** Unique associations were identified for TUD (baseline: $n_{Bonf}=2$, $n_{FDR}=21$; follow-up: $n_{Bonf}=3$, $n_{FDR}=34$), CUD (baseline: $n_{Bonf}=20$, $n_{FDR}=136$; follow-up: $n_{Bonf}=21$, $n_{FDR}=128$), and OUD PGS (baseline: $n_{Bonf}=0$, $n_{FDR}=0$; follow-up: $n_{Bonf}=2$, $n_{FDR}=12$), but not for PAU PGS, when the four non-ADDrf PGS were entered into the model simultaneously (**Supplemental Tables S13-14)**. CUD PGS was uniquely associated primarily with phenotypes indicating increased impulsivity and externalizing behaviors as measured on the CBCL, K-SADS, youth Brief Problem Monitor, Early Adolescent Temperament Questionnaire, and BIS/BAS. TUD PGS was uniquely linked with increased self-reported time spent on social media at baseline and with difficulty sticking to plans, energy/feelings commonly being at extreme ends, and social jetlag at follow-up. Finally, OUD PGS was independently associated with having more than one social media account and with a higher percent of individuals in one's neighborhood with a disability ($B \leq 0.032$, $p \leq 3.52e-05$, $p_{FDR} \leq 0.014$, $p_{Bonferroni} \leq 0.0497$).

**Phenotypic Pruning.** Pruning associations based on phenotypic correlation and strength of association (i.e., p-value) with the SUD PGS resulted in a largely domain non-specific monotonic reduction in significant associations. Associations observed to be independent at or below an $R^2$ threshold of 0.1 included 50%, 50%, 24%, 40%, and 71% of significant baseline associations for PAU, TUD, CUD, OUD, and ADDrf PGS, respectively, and 39%, 40%, 11%, 34%, and 53% of follow-up associations, respectively (**Supplemental Tables 9-10**). At baseline, these phenotypes included screentime and media use (mature-rated video game frequency, R-rated movie frequency, weekend social media frequency, total weekend screentime), family behavior and mental health (caregiver failure to pay debt, caregiver history of depression, paternal drug problems, caregiver somatic problems, caregiver rule-breaking behavior, caregiver history of anxiety, caregiver history of getting into trouble, and caregiver history of depression), prenatal/postnatal and early development (prenatal tobacco exposure and cigarettes/day before maternal knowledge of pregnancy, unplanned pregnancy, prenatal cannabis exposure before maternal knowledge of pregnancy), substance use and perceptions (total caffeine intake), substance-related environment (cigarette accessibility, different smoking rules in family, different cannabis rules in family), cognition (picture vocabulary, picture sequence memory test, total cognition composite, rey auditory verbal learning test, and list sorting working memory), parent educational attainment, and child behavior and mental health (total hours sleep per night, K-SADS past trauma-related concentration problems and exaggerated startle response, bis/bas fun-seeking, K-SADS depression-related guilt, K-SADS pressured speech, CBCL externalizing problems, CBCL spending time with peers who get into trouble, UPPS-P not getting things done on time, and talking in one's sleep).

At follow-up, these phenotypes included screentime and media use (weekday social media use, mature-rated video game frequency, R-rated movie frequency, using one's phone in the middle of the night, caregiver worry

that youth will post something inappropriate online, total weekday screentime, editing photos or videos to post on social media, only screen media seeming to help when having a bad day, watching/streaming movies/TV, having at least one social media account, having an internet-connected device or TV in one's bedroom), life events (new stepparent, family member having an SUD, sum of life events that affected the youth), substance use and perceptions (perceived smokeless tobacco use harm, perceived e-cigarette harm, expecting cannabis to be relaxing, energy drink intake, caffeine summary score), substance-related environment (number of friends who use "other" tobacco products), cognition (picture vocabulary), family environment (parent education, partner not youth's biological parent, caregiver-child conflict due to school problems, less frequent eating together as a family), peer environment (discrimination due to one's ethnicity, problems with bullying, involvement with peers who break rules, peers suspended from school, victim of gossip), school-related (detension/suspension, excused absences, drop in grades, disliking school, lower school environment), neighborhood/external environment (discrimination due to one's sexuality, discrimination due to one's weight, lower neighborhood high-school employment), physical health (past year number of sports and related activities), and child behavior and mental health (BIS/BAS going for something right away when one sees an opportunity to get something they want, self-reported disobedience at school, self-reported total problems, caregiver-reported destroying one's own things, CBCL DSM-5 conduct disorder, EATQ being scared of the dark at night, EATQ struggling to stick to a plan, EATQ lower inhibitory, one's feelings/energy usually up or down but rarely in the middle, social jetlag, number of times awake during the night, severity of psychotic-like experiences, suddenly not trusting others when seem to be watching/talking about them, number of hours reading per week for pleasure, number of hours' sleep per night, UPPS likes new/thrilling things even if they are a little scary).

**PGS-by-time moderation.** No PGS was significantly associated with change in any phenotype measured at both assessment waves, as indicated by a lack of significant wave-x-PGS interactions following Bonferroni or FDR correction (all $|B|\leq 0.37$, all p $\geq$9.84e-04, all $p_{FDR}\geq 0.45$, all $p_{Bonferroni}\geq 0.79$; **Supplemental Table 15**).

**Sex Moderation.** The significant interaction between PAU PGS and sex in predicting baseline ksads_9_37_p (caregiver-reported present active avoidance of phobic object; $B_{PGS}$=0.31, $B_{PGS}$*sex=-0.43, $p_{PGS}$*sex=3.77e-05, $p_{FDR\ PGS}$*sex=0.032, $p_{Bonferroni\ PGS}$*sex=0.038) involved a significant simple slope for female (B=0.31, p=5.12e-5) but not male participants (B=-0.12, p=-0.09). A Johnson-Neyman test indicated that the interaction was significant outside of the PAU PGS range -0.54 to 0.54 (**Supplemental Table S16; Extended Data Figure 4a**).

The significant interaction between CUD PGS and baseline pps_2_bother_yn (bothered by hearing strange sounds; $B_{PGS}$=0.40, $B_{PGS}$*sex=-0.66, $p_{PGS}$*sex=1.45e-05, $p_{FDR\ PGS}$*sex=0.015, $p_{Bonferroni\ PGS}$*sex=0.015) involved statistically significant simple slopes for female (B=0.41, p=2.16e-04) and male (B=-0.24, p=0.02) participants that were in the opposite direction. The Johnson Neyman interval for which the interaction was significant was outside of the CUD PGS range of -0.45 to 0.48 (**Supplemental Table S16; Supplemental Figure 4b**).

The significant interaction between TUD PGS and follow-up sleepdisturb5_p (feeling anxious when falling asleep; $B_{PGS}$=0.10, $B_{PGS}$*sex=-0.12, $p_{PGS}$*sex=1.45e-05, $p_{FDR\ PGS}$*sex=0.015, $p_{Bonferroni\ PGS}$*sex=0.015) involved a statistically significant simple slope for female (B=0.10, p=5.63e-06) but not male (B=-0.02, p=0.41)

participants. The Johnson Neyman interval for which the interaction was significant was outside of the TUD PGS range of -2.41 to -0.57 (**Supplemental Table S17; Extended Data Figure 4c**).

The significant interaction between OUD PGS and follow-up resiliency6b_y (number of close friends that are girls; $B_{PGS}$=-0.019, $B_{PGS}$*sex=0.10, $p_{PGS}$*sex=3.13e-05, $p_{FDR\ PGS}$*sex=0,043, $p_{Bonferroni\ PGS}$*sex=0.043) involved a statistically significant simple slope for male (B=0.08, p=3.22e-07) but not female (B=-0.02, p=0.26) participants. The Johnson Neyman interval for which the interaction was significant was outside of the range of observed data (**Supplemental Table S17; Extended Data Figure 4d**).

**Within-family PGS effects.** Following Bonferroni correction for the number of tests, within-family PGS effects were identified for PAU (baseline, n=1; follow-up, n=0), CUD (baseline, n=0; follow-up, n=5), and TUD (baseline, n=1; follow-up, n=1) PGS. Within-family PAU PGS was associated with increased caffeinated soda consumption (B=0.063, $p$=5.38e-05, $p_{FDR}$=1.29e-03, $p_{Bonferroni}$=1.29e-03) at baseline. Within-family TUD PGS was associated with increased caregiver self-reported restlessness at baseline (B=0.028, $p$=1.55e-03, $p_{FDR}$=0.028, $p_{Bonferroni}$=0.045) and increased caregiver-reported tendency for youth's moods to be generally "up or down, but rarely in the middle" on the General Behavior Inventory - Mania at two-year follow-up (B=0.055, $p$=7.82e-06, $p_{FDR}$=5.16e-04, $p_{Bonferroni}$=5.16e-04). Finally, within-family CUD PGS was associated with increased CBCL temper tantrums and CBCL breaking rules at school, home, or elsewhere; caregiver-child conflict; problems with bullying; and youth self-reported arguing (B≥0.075, $p$≤2.45e-04, $p_{FDR}$≤6.41e-03, $p_{Bonferroni}$≤0.032; **Supplemental Tables 18-19; Extended Data Figure 5**).

**PheWAS adjusted by phenotypic externalizing behavior.** The majority (PAU: 100%; TUD: 92.5%; CUD: 58.5%; OUD: 100%; ADDrf 100%) of Bonferroni-significant associations between SUD PGS and follow-up phenotypes remained significant after covarying for baseline CBCL externalizing problems and correcting for the number of tests (the number of Bonferroni-significant primary PheWAS associations, except CBCL externalizing problems) within each PGS (**Supplemental Table 20**).

The five phenotypes no longer associated with TUD PGS were related to externalizing (i.e., CBCL DSM-5 conduct problems and rulebreaking behavior, EATQ aggression and problems with inhibition) or sleep problems (i.e., total sleep disturbances score) and were not particularly strongly associated with TUD PGS in primary analyses (i.e., $p_{Bonferroni}$≥0.014). Social jetlag remained the strongest association, and phenotypes related to screentime and media use, caffeine intake, and school performance also continued to exhibit strong associations (**Supplemental Table 20**).

Of the 56 phenotypes no longer significantly associated with CUD PGS, 22 came from the CBCL and 11 from the EATQ (compared to four and two, respectively, that remained associated); many of these reflected externalizing, impulsive, and/or inattentive behavior. Most screentime and media use items continued to be associated with CUD PGS (i.e., 13 phenotypes representing time spent on various screen-related activities, having social media, and caregivers following their children on social media, compared to three phenotypes no longer associated with CUD PGS that reflected caregivers' reports of concern about, and motivations for, their child's

screen media use). The majority of youth self-report of behavior problems, school performance, social jetlag, stressful life events, psychotic-like events, school involvement, and caffeine intake phenotypes remained significantly associated with CUD PGS following adjustment for baseline CBCL externalizing problems and Bonferroni correction ((**Supplemental Table 20**).

***African-like Genetic Ancestry***

**Substance-naive PheWAS**. Though there were no significant associations observed in the primary PheWAS at baseline, results from the substance-naive sample are shown in **Supplemental Table 23**. All follow-up phenotypes associated with AUD and CUD PGS in primary analyses remained significantly associated with the PGS in the substance-naive subsample following Bonferroni correction for the number of tests (one and six, respectively; $|B|\geq 0.11$, $p\leq 3.92e-04$, $p_{Bonferroni}\leq 2.35e-03$; **Supplemental Table 24**).

**Simultaneous SUD PGS PheWAS.** No SUD PGS was independently associated with any phenotype at baseline when AUD, TUD, CUD, and OUD PGS were entered simultaneously into the model ($|B|\leq 0.25$, OR≤1.52, $p\geq 9.37e-05$, $p_{FDR}\geq 0.088$, $p_{Bonferroni}\geq 0.088$; **Supplemental Table 25**). CUD PGS was significantly independently associated with CBCL DSM-5 ADHD, attention problems, and disobedience at home ($B\geq 0.15$, $p\leq 1.52e-05$, $p_{FDR}\leq 6.88e-03$, $p_{Bonferroni}\leq 0.021$; **Supplemental Table 26**).

**Phenotypic Pruning.** Pruning associations with CUD PGS at follow-up based on phenotypic correlation and strength of association (i.e., p-value) showed that CBCL externalizing problems and DSM-5 ADHD had the strongest and most independent associations ($R^2=0.6$), followed by CBCL DSM-5 conduct disorder and total problems ($R^2=0.8$), CBCL attention problems ($R^2=0.9$), and aggressive problems (pruned immediately at $R^2=1.0$; **Supplemental Table 22**).

**PGS-by-time moderation.** No PGS was significantly associated with change in any phenotype measured at both assessment waves (all interaction $|B|\leq 0.37$, all $p\geq 1.16e-03$, all $p_{FDR}\geq 0.51$, all $p_{Bonferroni}\geq 0.72$; **Supplemental Table 27**).

**Sex Moderation.** No significant PGS*sex indirections were identified following Bonferroni correction (all $|B|\leq 0.67$, all $p\geq 1.72e-04$, all $p_{FDR}\geq 0.16$, all $p_{Bonferroni}\geq 0.19$; **Supplemental Tables 28-29).**

**Within-family PGS effects.** The single Bonferroni-significant AUD PGS association at follow-up was not significant at the within-family level ($B=0.015$, $p=0.78$). Within-family CUD PGS was positively associated with all six CBCL scales following FDR correction for six tests, and the association with the CBCL DSM-5 conduct disorder scale additionally survived Bonferroni correction ($B\geq 0.071$, $p\leq 0.030$, $p_{FDR}\leq 0.036$, $p_{Bonferroni}\geq 0.048$; **Supplemental Table 30**).

**PheWAS adjusted by phenotypic externalizing behavior.** AUD PGS remained significantly negatively associated with knowing how to get in touch with caregivers when they are not at home after covarying for baseline CBCL externalizing problems ($B=-0.12$, $p=2.77e-05$). CUD PGS remained significantly positively

associated with the CBCL DSM-5 conduct disorder scale (B=0.054, $p$=9.88e-03, $p_{\text{Bonferroni}}$=0.049) but not with the CBCL DSM-5 ADHD scale or CBCL aggressive, attention, or total problems (B≤0.050, $p$≥0.063, $p_{\text{Bonferroni}}$≥0.31; **Supplemental Table 31**).

***Indirect effects through neuroimaging-derived phenotypes.*** The brain-wide and phenome-wide significant imaging associations in individuals of European ancestry that were also observed ($p$<0.05) in the substance-naive sample were carried forward into indirect effects analyses. Following Bonferroni correction for the number of tests within each PGS (PAU PGS: 51 tests; TUD PGS: 67*3 brain regions = 201 tests; CUD PGS: 134*2 = 268 tests), 10, 5, and 11 associations between imaging metric and follow-up phenotype were identified for PAU, TUD, and CUD PGS, respectively (Supplemental Table 2.6.32). With the exception of RSFC-sensorimotor hand-sensorimotor mouth network indirectly linking PAU PGS to one's caregiver's partner *not* being a biological parent (|average causal mediation effect| = 5.04e-04, proportion mediated = 0.027, $p$=5.60e-03, $p_{Bonferron}$=0.056), all of these imaging metrics significantly indirectly linked the PGS to the respective follow-up phenotype following Bonferroni correction for all tests within each PGS (|average causal mediation effect| ≥ 1.34e-03, proportion mediated ≥ 0.0275, $p$≤5.40e-03, $p_{Bonferroni}$≤0.027[1]; **Supplemental Table 35; Extended Data Figures 8-10**).

***Cross-ancestry meta-analysis.***

**Baseline** (see **Supplemental Table 36**).

***Problematic Alcohol Use/Alcohol Use Disorder PGS.*** Of the 25 phenotypes measured in both genetic ancestry groups that were Bonferroni-significant in EUR, 7 were Bonferroni-significant in the cross-ancestry meta-analysis: Greater weekend social media hours, mature-rated video game frequency, R-rated movie frequency, total weekend screentime, prenatal cigarettes per day both before and after pregnancy knowledge, and unplanned pregnancy (Meta |B|≥0.023, OR=1.18, $p$≤2.96e-05, $p_{Bonferroni}$≤0.028; EUR |B|≥0.023, OR=1.21, $p$≤2.63e-05, $p_{Bonferroni}$≤0.033; AFR |B|≤0.021, OR=1.11, $p$≥5.67e-03, $p_{FDR}$≥0.92). An additional 3 phenotypes were Bonferroni-significant in EUR and FDR-significant in the meta-analysis: fewer hours of sleep per night, caregiver history of alcohol problems, and paternal history of alcohol problems (Meta B=0.059, ORs=1.28, $p_{FDR}$≤0.021, $p_{Bonferroni}$≥0.065; EUR B=0.089, OR≥1.35, $p$≤3.50e-05, $p_{Bonferroni}$≤0.045; AFR B=0.037, OR≤1.17, $p$≥0.089, $p_{FDR}$≥0.92). Four phenotypes were FDR-significant in EUR and Bonferroni-significant in the meta-analysis: BIS/BAS doing things on the spur of the moment, CBCL DSM-5 conduct disorder, lower caregiver educational attainment, and CBCL swearing/obscene language (Meta |B|≥0.024, $p$≤4.15e-03, $p_{Bonferroni}$≤0.046; EUR |B|≥0.026, $p$≤1.25e-04, $p_{FDR}$≤4.27e-03, $p_{Bonferroni}$≥0.066). Q-tests indicated that the ancestry-specific effect sizes for these phenotypes were not significantly heterogeneous (Q≤1.50, $Q_{Pval}$≥0.22) and that percent of the total variance attributed to heterogeneity (i.e., $I^2$) was between 0 and 33.3%. For the 11 phenotypes Bonferroni-significant in EUR but not significant following multiple testing correction in the meta-analysis, the Q-tests were largely significant (10 $Q_{Pval}$≤0.049, 1 $Q_{Pval}$=0.21), and $I^2$=36.9-91.5. These metrics of heterogeneity should be

[1] The mediation package's mediate() function outputs values of "0" for p-values that are less than 2.2e-16; for the purposes of multiple testing correction, these values were assigned a value of 2.2e-16, as also noted in Supplemental Table 35.

interpreted with caution, however, because only two effect sizes are contributing to each meta-regression; the Q-test may be underpowered, and the $I^2$ statistics may be biased in either direction(21,22).

***Tobacco Use Disorder PGS.*** Of the 30 phenotypes measured in both genetic ancestry groups that were Bonferroni-significant in EUR, 9 were Bonferroni-significant in the cross-ancestry meta-analysis: Prenatal tobacco exposure and cigarettes/day pre pregnancy knowledge and prenatal tobacco exposure after pregnancy knowledge, caregiver self-reported failure to pay debts/other financial obligations, lower NIH Toolbox list sorting working memory, fewer hours of sleep per night, BIS/BAS fun-seeking, CBCL lying/cheating, and CBCL DSM-5 conduct disorder (Meta $|B|\geq 0.029$, OR≥1.41, $p\leq$2.26e-05, $p_{Bonferroni}\leq 0.021$; EUR $|B|\geq 0.030$, OR≥1.46, $p\leq$3.25e-05, $p_{Bonferroni}\leq 0.041$; AFR $|B|\leq 0.076$, OR≤1.37, $p\geq$8.74e-04, $p_{FDR}\geq 0.61$). An additional 7 phenotypes were FDR-significant in EUR and Bonferroni-significant in the meta-analysis: Prenatal cannabis exposure pre pregnancy knowledge, maternal history of depression, past-year detention or suspension, greater cigarette accessibility, greater UPPS-P positive urgency, and greater CBCL rule-breaking and externalizing problems (Meta $|B|\geq 0.031$, OR≥1.18, $p\leq$3.04e-03, $p_{Bonferroni}\leq 0.049$; EUR $|B|\geq 0.033$, OR≥1.18, $p\leq$1.42e-03, $p_{FDR}\leq 0.017$, $p_{Bonferroni}\geq 0.13$; AFR $|B|\leq 0.059$, OR≤1.28, $p\geq$8.77e-03, $p_{FDR}\geq 0.61$). Q-tests indicated that the ancestry-specific effect sizes for these phenotypes were not significantly heterogeneous (Q≤1.61, $Q_{Pval}\geq 0.20$) and that percent of the total variance attributed to heterogeneity (i.e., $I^2$) was between 0 and 37.9%. For the 21 phenotypes Bonferroni-significant in EUR but not significant following multiple testing correction in the meta-analysis, the Q-tests were largely significant (15 $Q_{Pval}\leq 0.030$, 6 $Q_{Pval}$=0.057-0.10), and $I^2$=62.4-94.4; but should be interpreted with caution.

***Cannabis Use Disorder PGS.*** Of the 70 phenotypes measured in both genetic ancestry groups that were Bonferroni-significant in EUR, 45 were Bonferroni-significant in the cross-ancestry meta-analysis. These included CBCL scales (externalizing, total problems, aggressive, attention problems, social problems, DSM-5 conduct disorder, DSM-5 oppositional defiant disorder, DSM-5 ADHD, DSM-5 depressive disorder, and the stress scale) and 12/15 CBCL items, 12/13 KSADS ADHD and ODD items, UPPS-P difficulty getting things done on time, the PGMI mania summary score, prenatal tobacco and cannabis exposure pre pregnancy knowledge (but not unplanned pregnancy), greater total sleep problems (but not hours per day), greater cigarette accessibility, and caregiver self-reported rule-breaking (Meta $|B|\geq 0.032$, OR≥1.17, $p\leq$3.88e-05, $p_{Bonferroni}\leq 0.037$; EUR $|B|\geq 0.030$, OR≥1.17, $p\leq$3.36e-05, $p_{Bonferroni}\leq 0.043$; AFR $|B|\leq 0.10$, OR≤1.28, $p\geq$1.49e-04, $p_{FDR}\geq 0.063$). An additional 8 phenotypes were Bonferroni-significant in EUR and FDR-significant in the meta-analysis: 2 CBCL items (demands attention, disobedient in school), teacher-rated difficulties concentrating, disorders of initiating and maintaining sleep, lower school performance, caregiver history of depression and paternal history of drug problems, and lower caregiver education (Meta $|B|\geq 0.033$, OR≥1.27, $p\leq$9.96e-03, $p_{FDR}\leq 0.041$, $p_{Bonferroni}\geq 0.12$; EUR $|B|\geq 0.034$, OR≥1.36, $p\leq$1.43e-05, $p_{Bonferroni}\leq 0.018$; AFR $|B|\leq 0.069$, OR≤1.18, $p\geq$9.86e-03, $p_{FDR}\geq 0.18$). Twenty five phenotypes were FDR-significant in EUR and Bonferroni-significant in the meta-analysis: CBCL thought problems and 5 CBCL items related to externalizing and attention problems, 11 KSADS items related to ADHD and ODD, K-SADS past distractibility on the Bipolar Disorders module, greater severity of psychotic-like experiences, 2 teacher-reported items related externalizing and attention problems, prenatal cannabis exposure quantity pre pregnancy knowledge, caregiver-child conflict and conflict related to youth's moods, and poorer grades in school (Meta $|B|\geq 0.019$, OR≥1.13, $p\leq$5.24e-05, $p_{Bonferroni}\leq 0.0489$; EUR $|B|\geq 0.018$, OR≥1.13

$p \leq 1.38e-03$, $p_{FDR} \leq 9.11e-03$, $p_{Bonferroni} \geq 0.063$; AFR $|B| \leq 0.088$, OR≤1.28, $p \geq 2.04e-03$, $p_{FDR} \geq 0.11$). Q-tests indicated that the ancestry-specific effect sizes for these phenotypes were not significantly heterogeneous (Q≤1.87, $Q_{Pval} \geq 0.17$) and that percent of the total variance attributed to heterogeneity (i.e., $I^2$) was between 0 and 46.6%. For the 19 phenotypes Bonferroni-significant in EUR but not significant following multiple testing correction in the meta-analysis, the Q-tests were largely significant (13 $Q_{Pval} \leq 0.041$, 6 $Q_{Pval}$=0.050-0.13), and $I^2$=56.1-90.9; but should be interpreted with caution.

***Opioid Use Disorder PGS***. Of the 10 phenotypes measured in both genetic ancestry groups that were Bonferroni-significant in EUR, 2 were Bonferroni-significant in the cross-ancestry meta-analysis: Caffeine intake summary score and caffeinated soda intake (Meta B≥0.047, $p \leq 4.79e-06$, $p_{Bonferroni} \leq 4.50e-03$; EUR B≥0.050, $p \leq 1.62e-05$, $p_{Bonferroni} \leq 0.021$; AFR B≤0.054, $p \geq 0.017$, $p_{FDR} \geq 0.99$). An additional 2 phenotypes were Bonferroni-significant in EUR and FDR-significant in the meta-analysis: NIH Toolbox picture sequence memory test and picture vocabulary test (Meta B≤-0.049, $p \leq 8.31e-04$, $p_{FDR} \leq 0.049$, $p_{Bonferroni} \geq 0.17$; EUR B≤-0.059, $p \leq 8.42e-06$, $p_{Bonferroni} \leq 0.011$; AFR $|B| \geq 0.026$, $p \geq 0.25$). Prenatal tobacco exposure pre pregnancy knowledge was further Bonferroni-significant in the meta-analysis and FDR-significant in EUR (Meta OR=1.25, $p$=2.02e-05, $p_{Bonferroni}$=0.019; EUR OR=1.28, $p$=2.26e-04, $p_{FDR}$=0.012, $p_{Bonferroni}$=0.29; AFR OR=1.20, $p$=0.025, $p_{FDR}$=0.99). Q-tests indicated that the ancestry-specific effect sizes for these phenotypes were not significantly heterogeneous (Q≤1.34, $Q_{Pval} \geq 0.25$) and that percent of the total variance attributed to heterogeneity (i.e., $I^2$) was between 0 and 25.6%. For the 6 phenotypes Bonferroni-significant in EUR but not significant following multiple testing correction in the meta-analysis, the Q-tests were largely significant (5 $Q_{Pval} \leq 0.026$, 1 $Q_{Pval}$=0.081), and $I^2$=67.2-92.7; but should be interpreted with caution.

***Addiction Risk Factor PGS***. Of the 16 phenotypes measured in both genetic ancestry groups that were Bonferroni-significant in EUR, 5 were Bonferroni-significant in the cross-ancestry meta-analysis: BIS/BAS fun-seeking and doing things in the spur of the moment, total weekend screentime and R-rated movie frequency, and prenatal cigarettes/day pre pregnancy knowledge (Meta B≥0.028, $p \leq 3.02e-05$, $p_{Bonferroni} \leq 0.028$; EUR B≥0.032, $p \leq 3.65e-05$, $p_{Bonferroni} \leq 0.046$; AFR B≤0.049, $p \geq 0.054$). One phenotype (unplanned pregnancy) was additionally Bonferroni-significant in EUR and FDR significant in the meta-analysis (Meta OR=1.17, $p$=1.18e-03, $p_{FDR}$=0.030, $p_{Bonferroni}$=1.00; EUR OR=1.21, $p$=1.95e-07, $p_{Bonferroni}$=2.47e-04; AFR OR=1.10, $p$=0.17). Q-tests indicated that the ancestry-specific effect sizes for these phenotypes were not significantly heterogeneous (Q≤1.77, $Q_{Pval} \geq 0.18$) and that percent of the total variance attributed to heterogeneity (i.e., $I^2$) was between 0 and 43.5%. For the 10 phenotypes Bonferroni-significant in EUR but not significant following multiple testing correction in the meta-analysis, 3 Q-tests were significant ($Q_{Pval} \leq 0.038$) and 7 were not ($Q_{Pval}$=0.098-0.23), and $I^2$=30.7-87.7; but should be interpreted with caution.

**Follow-up** (see **Supplemental Table 37**).

***Problematic Alcohol Use/Alcohol Use Disorder PGS.*** Of the 50 phenotypes measured in both genetic ancestry groups that were Bonferroni-significant in EUR, 9 were Bonferroni-significant in the cross-ancestry meta-analysis: Total "bad" life events, the summary score of the degree to which "bad" life events affected the youth,

CBCL DSM-5 conduct disorder and rule-breaking behavior, youth-reported total problems, psychotic-like experiences severity score, social media frequency, using one's phone/device in the middle of the night, and spending time with peers who have been suspended (Meta $B \geq 0.044$, $p \leq 2.25e\text{-}05$, $p_{Bonferroni} \leq 0.031$; EUR $B \geq 0.047$, $p \leq 1.32e\text{-}05$, $p_{Bonferroni} \leq 0.022$; AFR $B \leq 0.068$, $p \geq 0.011$, $p_{Bonferroni} = 1.00$). An additional 5 phenotypes were Bonferroni-significant in EUR and FDR-significant in the meta-analysis: Total number of life events and the summary score of the degree to which the youth was affected by life events, lower scores on the NIH Toolbox Picture Vocabulary Test, UPPS-P liking new, thrilling things, and greater weekend social media hours (Meta $|B| \geq 0.050$, $p \leq 2.96e\text{-}03$, $p_{FDR} \leq 0.045$, $p_{Bonferroni} \geq 0.077$; EUR $|B| \geq 0.060$, $p \leq 2.91e\text{-}05$, $p_{Bonferroni} \leq 0.049$; AFR $|B| \leq 0.039$, $p \geq 0.16$). Seven phenotypes were FDR-significant in EUR and Bonferroni-significant in the meta-analysis: Youth-reported externalizing and attention problems, lower EAT attention and effortful control, CBCL destroying one's own things, not getting along with one's teachers, and being chased by another youth as though they were trying to hurt them (Meta $|B| \geq 0.026$, $p \leq 3.66e\text{-}05$, $p_{Bonferroni} \leq 0.0497$; EUR $|B| \geq 0.027$, $p \leq 3.09e\text{-}04$, $p_{FDR} \leq 5.58e\text{-}03$, $p_{Bonferroni} \geq 0.062$; AFR $|B| \leq 0.061$, $p \geq 0.042$, $p_{FDR} \geq 0.87$). Q-tests indicated that the ancestry-specific effect sizes for these phenotypes were not significantly heterogeneous ($Q \leq 1.80$, $Q_{Pval} \geq 0.18$) and that percent of the total variance attributed to heterogeneity (i.e., $I^2$) was between 0 and 44.5%. For the 36 phenotypes Bonferroni-significant in EUR but not significant following multiple testing correction in the meta-analysis, the majority of Q-tests were significant (23 $Q_{Pval} \leq 0.048$, 13 $Q_{Pval} = 0.056\text{-}0.21$), and $I^2 = 35.6\text{-}93.0$, but should be interpreted with caution.

***Tobacco Use Disorder PGS.*** Of the 67 phenotypes measured in both genetic ancestry groups that were Bonferroni-significant in EUR, 23 were Bonferroni-significant in the cross-ancestry meta-analysis. These included CBCL externalizing scales and items (externalizing, DSM-5 conduct disorder, rule-breaking problems, destroying one's own things, and lying/cheating); youth-reported attention problems and having a hot temper, and teacher-reported total problems; EATQ scales and items (aggression, attention, putting off working, slamming the door when angry, and being rude), total sleep problems, poorer grades in school, spending time with peers who have been suspended, having experienced reputational victimization, social media frequency, having a family member with an SUD, coffee consumption, and lower perceived harm of regular e-cigarette and regular smokeless tobacco use (Meta $|B| \geq 0.031$; OR=1.23, $p \leq 8.87e\text{-}06$, $p_{Bonferroni} \leq 0.012$; EUR $|B| \geq 0.033$, OR=1.25, $p \leq 2.63e\text{-}05$, $p_{Bonferroni} \leq 0.045$; AFR $|B| \leq 0.076$, OR=1.17, $p \geq 0.010$, $p_{FDR} \geq 0.53$). An additional 5 phenotypes were Bonferroni-significant in EUR and FDR-significant in the meta-analysis: The summary score of the degree to which the youth was affected by life events, lower EATQ effortful control, creator caffeine summary score, mature-rated video game frequency, and having experienced detention or suspension (Meta $|B| \geq 0.057$, OR=1.29, $p \leq 3.32e\text{-}03$, $p_{FDR} \leq 0.031$, $p_{Bonferroni} \geq 0.16$; EUR $|B| \geq 0.067$, OR=1.37, $p \leq 4.14e\text{-}06$, $p_{Bonferroni} \leq 7.03e\text{-}03$; $|B| \leq 0.036$, OR=1.20, $p \geq 0.028$, $p_{FDR} \geq 0.64$). Nine phenotypes were FDR-significant in EUR and Bonferroni-significant in the meta-analysis: CBCL aggressive problems and lacking guilt after misbehaving and having sudden changes in one's mood, youth-reported trouble concentrating and sitting still, EATQ greater negative affect and getting sidetracked, UPPS-P acting without thinking when happy, and being the victim of rumors (Meta $|B| \geq 0.034$, $p \leq 3.63e\text{-}05$, $p_{Bonferroni} \leq 0.049$; EUR $|B| \geq 0.032$, $p \leq 2.99e\text{-}04$, $p_{FDR} \leq 4.14e\text{-}03$, $p_{Bonferroni} \geq 0.056$; AFR $|B| \leq 0.068$, $p \geq 0.013$, $p_{FDR} \geq 0.54$). Q-tests indicated that the ancestry-specific effect sizes for these phenotypes were not significantly heterogeneous ($Q \leq 2.17$, $Q_{Pval} \geq 0.14$) and that percent of the total variance

attributed to heterogeneity (i.e., $I^2$) was between 0 and 53.9%. For the 39 phenotypes Bonferroni-significant in EUR but not significant following multiple testing correction in the meta-analysis, there was evidence of heterogeneity in effect sizes (12 $Q_{Pval}$≤0.041, 27 $Q_{Pval}$=0.053-0.21; $I^2$=35.4-94.1; but these should be interpreted with caution).

***Cannabis Use Disorder PGS.*** Of the 133 phenotypes measured in both genetic ancestry groups that were Bonferroni-significant in EUR, 58 were Bonferroni-significant in the cross-ancestry meta-analysis. These included higher CBCL externalizing, attention, rule-breaking, and aggressive problems, CBCL DSM-5 oppositional defiant and conduct disorder scales, and 14 individual CBCL items loading onto these scales; lower EATQ effortful control, inhibitory control, attention, and activation scales, and 8 EATQ items reflecting distractibility, impulsivity, irritability, and procrastination; greater UPPS-P positive and negative urgency and two UPPS-P items; BIS/BAS going for what one wants right away; youth self-reported tendency to argue a lot and feeling worthless; teacher-reported failure to finish things once started; caregiver-reported general behavior inventory summary score and items reflecting rapid mood shifts and irritability alongside happiness and energy; total number and severity of psychotic-like experiences; greater total sleep problems, disorders of initiating and maintaining sleep, and fewer total hours of sleep per night; concentrating too much on parts over the whole; caregiver-youth conflict and conflict related to moods; lower school performance; caregiver self-reported rule-breaking; overt aggression; and having a family member with an SUD (Meta |B|≥0.033, OR≥1.19, *p*≤2.81e-05; $p_{Bonferroni}$≤0.0381; EUR |B|≥0.035, OR≥1.18, *p*≤2.74e-05, $p_{Bonferroni}$≤0.047). Or these, the CBCL externalizing, aggressive, and DSM-5 conduct disorder scales were also significantly associated with $CUD_{PGS}$ in AFR following Bonferroni correction (B≥0.12, *p*≤2.72e-05, $p_{Bonferroni}$≤0.037, and CBCL DSM-5 oppositional defiant disorder and rule-breaking scales and an item about rule-breaking were significant following FDR correction (B≥0.14, *p*≤2.15e-04, $p_{FDR}$≤0.43, $p_{Bonferroni}$≥0.19; all other |B|≤0.11, OR≤1.24, *p*≥1.01e-03, $p_{FDR}$≥0.077). Three phenotypes were Bonferroni-significant in EUR and AFR and FDR-significant in the meta-analysis: CBCL DSM-5 ADHD, attention problems, and total problems (Meta B≥0.11, *p*≤4.91e-03, $p_{FDR}$≤0.029, $p_{Bonferroni}$≥0.097; EUR B≥0.083, *p*≤2.30e-07, $p_{Bonferroni}$≤3.90e-04; AFR B≥0.14, *p*≤3.44e-05, $p_{Bonferroni}$≤0.047). Two associations were Bonferoni-significant in EUR and FDR-significant in AFR and the meta-analysis: higher CBCL stress scale score and disobedience in one's home (Meta B≥0.091, *p*≤2.25e-03, $p_{FDR}$≤0.015, $p_{Bonferroni}$≥0.082; EUR B≥0.068, *p*≤1.64e-07, $p_{Bonferroni}$≤0.041; AFR B≥0.13, *p*≤2.14e-04, $p_{FDR}$≤0.024, $p_{Bonferroni}$≥0.059). Ten associations were Bonferroni-significant in EUR, FDR-significant in the meta-analysis, and not significant following multiple testing correction in AFR, including two CBCL items reflecting externalizing behavior; youth-reported attention problems; poor school grades and having been suspended or sent to detention; spending time with peers who have been suspended; and the total number of bad life events (Meta B≥0.036, OR≥1.20, *p*≤4.79e-03, $p_{FDR}$≤0.029, $p_{Bonferroni}$≥0.075; EUR B≥0.031, OR≥1.27, *p*≤2.71e-06, $p_{Bonferroni}$≤4.61e-03; AFR B≤0.053, OR≤1.22, *p*≥1.25e-03, $p_{FDR}$≥0.077). Twenty phenotypes demonstrated Bonferroni-significant associations in the meta-analysis and FDR-significant associations in EUR, but non-significant associations following multiple testing correction in AFR (Meta B≥0.032, OR≥1.17, *p*≤3.35e-05, $p_{Bonferroni}$≤0.046; EUR B≥0.029, OR≥1.16 , *p*≤1.49e-03, $p_{FDR}$≤8.32e-03, $p_{Bonferroni}$≥0.053; AFR B≤0.099, OR≤1.43, *p*≥1.64e-03, $p_{FDR}$≥0.089). Q-tests indicated that the ancestry-specific effect sizes for these phenotypes were not significantly heterogeneous (92 Q≤2.94, $Q_{Pval}$≥0.087; 1 Q=4.88, $Q_{Pval}$=0.027) and that percent of the total variance attributed to heterogeneity (i.e., $I^2$) was between 0 and

79.5%. For the 60 phenotypes Bonferroni-significant in EUR but not significant following multiple testing correction in the meta-analysis, there was evidence of heterogeneity in effect sizes (48 $Q_{Pval} \leq 0.048$, 12 $Q_{Pval}=0.050$-0.16; $I^2=48.8$-95.0; but these should be interpreted with caution).

***Opioid Use Disorder PGS.*** Of the 35 phenotypes measured in both genetic ancestry groups that were Bonferroni-significant in EUR, two were Bonferroni-significant in the cross-ancestry meta-analysis: Youth self-reported tendency to argue a lot, and frequency of talking or texting on the phone (Meta $B \geq 0.060$, $p \leq 1.56\text{e-}05$, $p_{Bonferroni} \leq 0.021$; EUR $B \geq 0.066$, $p \leq 2.85\text{e-}06$, $p_{Bonferroni} \leq 4.84\text{e-}03$; AFR $B \leq 0.034$, $p \geq 0.24$). An additional 5 phenotypes were Bonferroni-significant in EUR and FDR-significant in the meta-analysis: Greater caffeine use summary score, greater neighborhood socioeconomic social vulnerability scores, lower neighborhood social and economic child opportunity index scores, lower scores on the NIH Toolbox picture vocabulary test, and lower perceived harm for regular smokeless tobacco use (Meta $|B| \geq 0.054$, $p \leq 5.85\text{e-}04$, $p_{FDR} \leq 0.038$, $p_{Bonferroni} \geq 0.091$; EUR $|B| \geq 0.063$, $p \leq 2.24\text{e-}05$, $p_{Bonferroni} \leq 0.038$; AFR $|B| \leq 0.046$, $p \geq 0.17$). Five phenotypes were significant following Bonferroni correction in the meta-analysis and FDR correction in EUR: lower neighborhood per capita income, greater neighborhood child opportunity index housing public assistance, lower neighborhood child opportunity index economic resource index, and greater caffeinated soda intake (Meta $|B| \geq 0.047$, $p \leq 2.58\text{e-}05$, $p_{Bonferroni} \leq 0.035$; EUR $|B| \geq 0.047$, $p \leq 2.93\text{e-}04$, $p_{FDR} \leq 6.99\text{e-}03$, $p_{Bonferroni} \geq 0.085$; AFR $|B| \leq 0.074$, $p \geq 0.015$, $p_{FDR} \geq 0.996$). Q-tests indicated that the ancestry-specific effect sizes for these phenotypes were not significantly heterogeneous ($Q \leq 1.36$, $Q_{Pval} \geq 0.24$) and that percent of the total variance attributed to heterogeneity (i.e., $I^2$) was between 0 and 26.5%. For the 28 phenotypes Bonferroni-significant in EUR but not significant following multiple testing correction in the meta-analysis, there was some evidence of heterogeneity in effect sizes (13 $Q_{Pval} \leq 0.045$, 15 $Q_{Pval}=0.073$-0.24; $I^2=26.9$-89.5; but these should be interpreted with caution).

***Addiction Risk Factor PGS.*** Of the 18 phenotypes measured in both genetic ancestry groups that were Bonferroni-significant in EUR, eight were Bonferroni-significant in the cross-ancestry meta-analysis: Greater maximum caffeine intake, total number of life events, sum of life events that affected the youth, total number of bad life events, sum of life events that affected youth in a bad way, teacher-reported school disobedience, social media use frequency, and having a TV or internet in one's bedroom (Meta $B \geq 0.043$, OR=1.15, $p \leq 8.95\text{e-}06$, $p_{Bonferroni} \leq 0.012$; EUR $B \geq 0.043$, OR=1.16, $p \leq 1.78\text{e-}05$, $p_{Bonferroni} \leq 0.030$; AFR $B \leq 0.071$, OR=1.11, $p \geq 0.011$, $p_{FDR} \geq 0.20$). Seven phenotypes were significant following Bonferroni correction in the meta-analysis and FDR correction in EUR: greater CBCL lying/cheating, waking up frequently during the night, mature-rated video game frequency and weekday screentime, and spending time with peers who have been suspended and are rule-breaking, as well as lower school grades (Meta $|B| \geq 0.036$, $p \leq 3.44\text{e-}05$, $p_{Bonferroni} \leq 0.047$; EUR $|B| \geq 0.036$, $p \leq 4.17\text{e-}04$, $p_{FDR} \leq 0.012$, $p_{Bonferroni} \geq 0.71$; AFR $|B| \leq 0.072$, $p \geq 9.48\text{e-}03$, $p_{FDR} \geq 0.20$). Q-tests indicated that the ancestry-specific effect sizes for these phenotypes were not significantly heterogeneous ($Q \leq 0.94$, $Q_{Pval} \geq 0.33$) and that percent of the total variance attributed to heterogeneity (i.e., $I^2$) was 0 for all 15 effects. For the 10 phenotypes Bonferroni-significant in EUR but not significant following multiple testing correction in the meta-analysis, there was some evidence of heterogeneity in effect sizes (6 $Q_{Pval} \leq 0.047$, 4 $Q_{Pval}=0.075$-0.18; $I^2=45.7$-89.0; but these should be interpreted with caution).

## References


1. Baurley JW, Edlund CK, Pardamean CI, Conti DV, Bergen AW (2016): Smokescreen: a targeted genotyping array for addiction research. *BMC Genomics* 17: 145.
2. Lam M, Awasthi S, Watson HJ, Goldstein J, Panagiotaropoulou G, Trubetskoy V, *et al.* (2020): RICOPILI: Rapid Imputation for COnsortias PIpeLIne. *Bioinformatics* 36: 930–933.
3. Taliun D, Harris DN, Kessler MD (2021): Sequencing of 53,831 diverse genomes from the NHLBI TOPMed Program. *Nature* 590: 290–299.
4. Ge T, Chen CY, Ni Y, Feng YCA, Smoller JW (2019): Polygenic prediction via Bayesian regression and continuous shrinkage priors. *Nat Commun* 10. https://doi.org/10.1038/s41467-019-09718-5
5. Ruan Y, Lin Y-F, Feng Y-CA, Chen C-Y, Lam M, Guo Z, *et al.* (2022): Improving polygenic prediction in ancestrally diverse populations [no. 5]. *Nat Genet* 54: 573–580.
6. Chang CC, Chow CC, Tellier LC, Vattikuti S, Purcell SM, Lee JJ (2015): Second-generation PLINK: rising to the challenge of larger and richer datasets. *GigaScience* 4: 7.
7. Miller AP, Baranger DAA, Paul SE, Garavan H, Mackey S, Tapert SF, *et al.* (2024): Neuroanatomical Variability and Substance Use Initiation in Late Childhood and Early Adolescence. *JAMA Netw Open* 7: e2452027.
8. Paul SE, Colbert SMC, Gorelik AJ, Johnson EC, Hatoum AS, Baranger DAA, *et al.* (2024): A phenome-wide association study of cross-disorder genetic liability in youth genetically similar to individuals from European reference populations. *Nat Ment Health* 2: 1327–1341.
9. Bates D, Mächler M, Bolker B, Walker S (2015): Fitting Linear Mixed-Effects Models Using lme4. *J Stat Softw* 67. https://doi.org/10.18637/jss.v067.i01
10. Bartoń K (2009): MuMIn: Multi-model inference, R package version 0.12.0 [R package version 0.12.0.].
11. Keller MC (2014): Gene × environment interaction studies have not properly controlled for potential confounders: the problem and the (simple) solution. *Biol Psychiatry* 75: 18–24.
12. Long JA (2019, February 19): interactions: Comprehensive, User-Friendly Toolkit for Probing Interactions. p 1.2.0.

13. Gorelik AJ, Paul SE, Miller AP, Baranger DAA, Lin S, Zhang W, *et al.* (2024): Associations between polygenic scores for cognitive and non-cognitive factors of educational attainment and measures of behavior, psychopathology, and neuroimaging in the adolescent brain cognitive development study. *Psychol Med* 54: 1–15.

14. Paul SE, Elsayed NM, Colbert SMC, Bogdan R, Hatoum AS, Barch DM (2024): Family income and polygenic scores are independently but not interactively associated with cognitive performance among youth genetically similar to European reference populations. *Dev Psychopathol* 1–15.

15. Selzam S, Ritchie SJ, Pingault J-B, Reynolds CA, O'Reilly PF, Plomin R (2019): Comparing Within- and Between-Family Polygenic Score Prediction. *Am J Hum Genet* 105: 351–363.

16. Kotov R, Krueger RF, Watson D, Cicero DC, Conway CC, DeYoung CG, *et al.* (2021): The Hierarchical Taxonomy of Psychopathology (HiTOP): A Quantitative Nosology Based on Consensus of Evidence. *Annu Rev Clin Psychol* 17: 83–108.

17. Karlsson Linnér R, Mallard TT, Barr PB, Sanchez-Roige S, Madole JW, Driver MN, *et al.* (2021): Multivariate analysis of 1.5 million people identifies genetic associations with traits related to self-regulation and addiction [no. 10]. *Nat Neurosci* 24: 1367–1376.

18. Kendler KS, Prescott CA, Myers J, Neale MC (2003): The Structure of Genetic and Environmental Risk Factors for Common Psychiatric and Substance Use Disorders in Men and Women. *Arch Gen Psychiatry* 60: 929–937.

19. Tingley D, Yamamoto T, Hirose K, Keele L, Imai K (2014): mediation: R Package for Causal Mediation Analysis. *J Stat Softw* 59: 1–38.

20. Viechtbauer W (2010): Conducting Meta-Analyses in R with the metafor Package. *J Stat Softw* 36. https://doi.org/10.18637/jss.v036.i03

21. von Hippel PT (2015): The heterogeneity statistic I2 can be biased in small meta-analyses. *BMC Med Res Methodol* 15: 35.

22. Huedo-Medina TB, Sánchez-Meca J, Marín-Martínez F, Botella J (2006): Assessing heterogeneity in meta-analysis: Q statistic or I2 index? *Psychol Methods* 11: 193–206.

## Supplemental Figure 1.

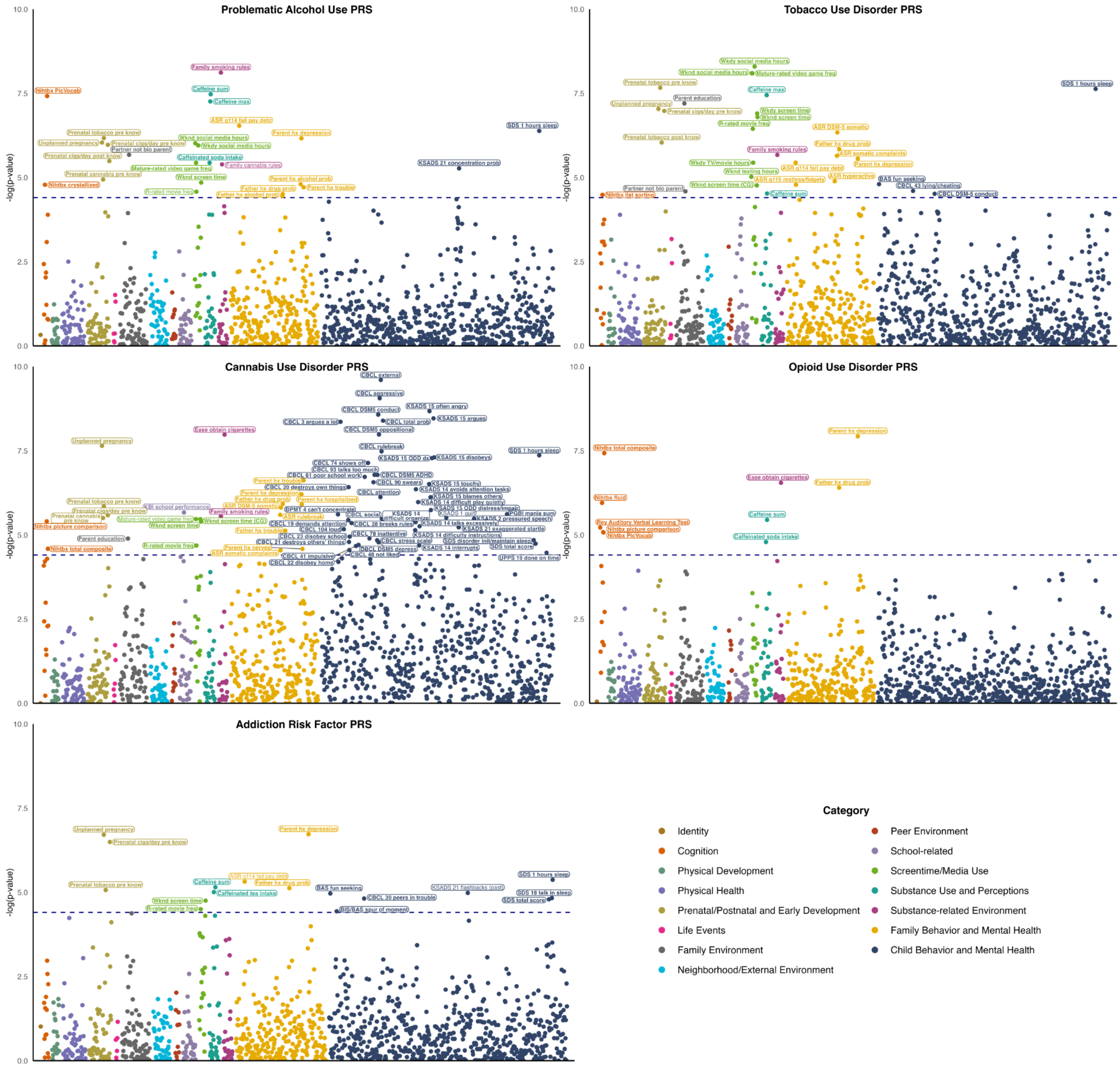


*Note*. Manhattan plots depicting primary PheWAS associations at the baseline wave among individuals genetically similar to European reference populations. The 1,271 examined phenotypes are spread along the x-axis, & the y-axes represent the -log$_{10}$ of the uncorrected *p* values of associations between each PGS & each phenotype. The points are color-coded by phenotype domain. The dashed lines indicate the Bonferroni-significant threshold (i.e., -log$_{10}$(0.05/1271) = 4.405). Full results are located in **Supplemental Table 9**.

**Supplemental Figure 2.**

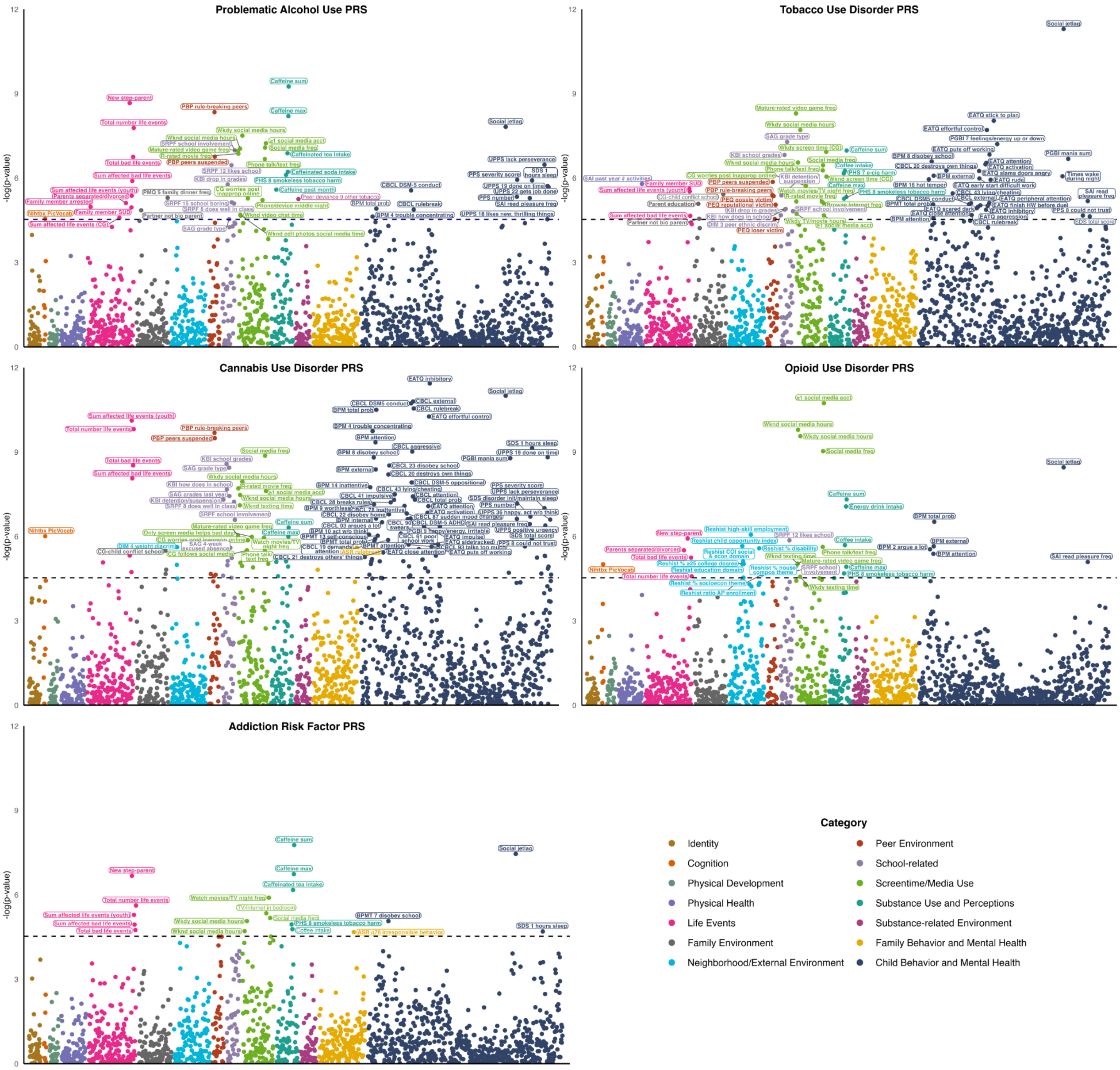


*Note*. Manhattan plots depicting primary PheWAS associations at the two-year follow-up wave among individuals genetically similar to European reference populations. The 1,697 examined phenotypes are spread along the x-axis, & the y-axes represent the -log$_{10}$ of the uncorrected *p* values of associations between each PGS & each phenotype. The points are color-coded by phenotype domain. The dashed lines indicate the Bonferroni-significant threshold (i.e., -log$_{10}$(0.05/1697) = 4.53). Full results are located in **Supplemental Table 10**.

**Supplemental Figure 3.**

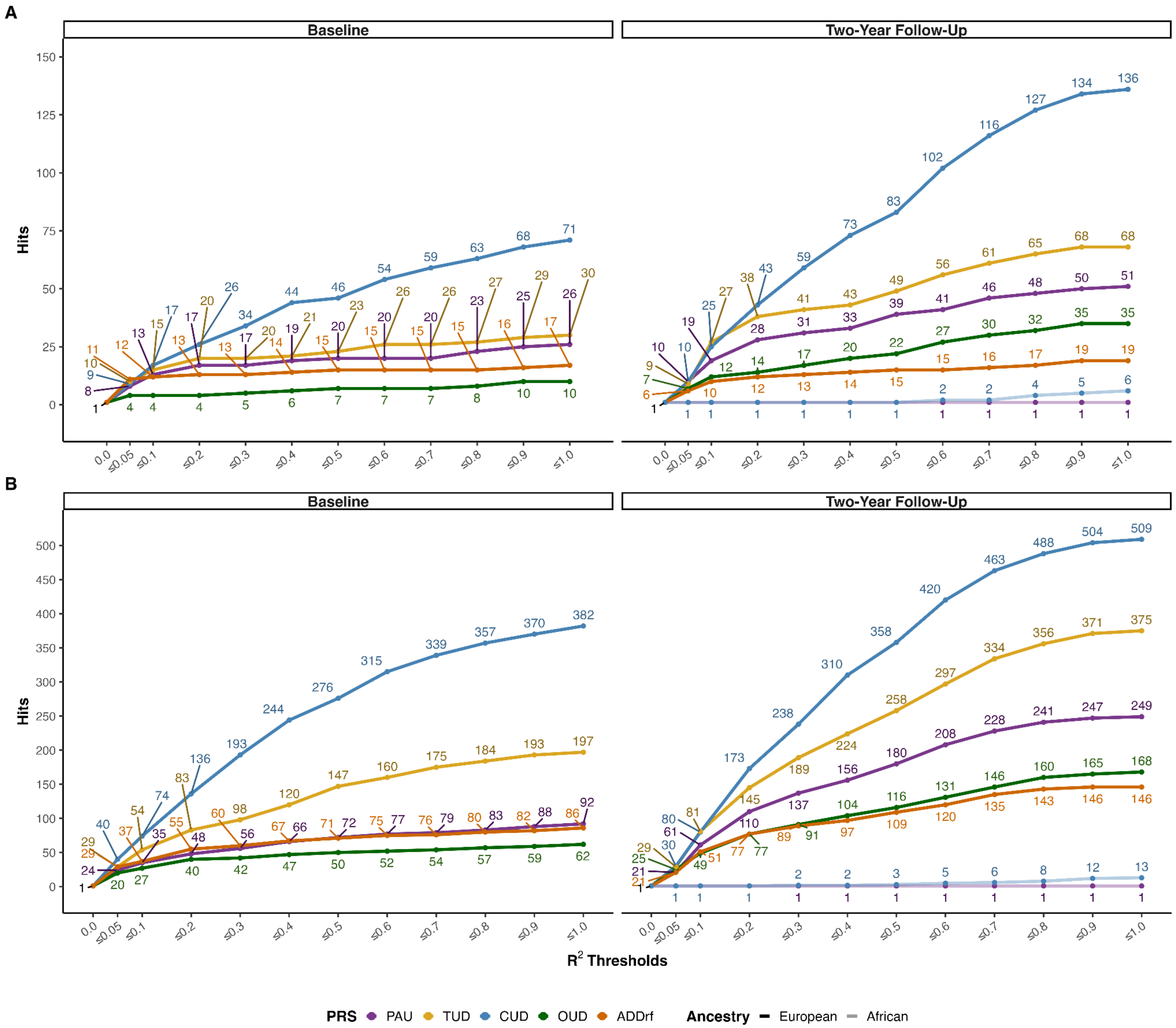


Pruning significant associations according to correlations among phenotypes & strength of association between each phenotype & each PGS. Pseudo $R^2$ values were obtained for each pair of phenotypes significantly (**A**, Bonferroni-corrected; **B**, FDR-corrected) associated with each PGS. At each $R^2$ threshold, the phenotype with the higher (i.e., less significant) p-value within each phenotype pair whose $R^2$ was above the threshold was removed. The following $R^2$ thresholds were examined: 0.05, 0.1, 0.2, 0.3, 0.4, 0.5, 0.6, 0.7, 0.8, 0.9, 1.0, with 1.0 including all significantly associated phenotypes. Colors denote different PGS. More transparent lines denote associations in individuals most genetically similar to African reference populations; these are only included in the Two-Year Follow-Up plots given the lack of significant associations observed at Baseline. PAU = Problematic Alcohol Use; TUD = Tobacco Use Disorder; CUD = Cannabis Use Disorder; OUD = Opioid Use Disorder; ADDrf = Addiction Risk Factor; AUD = Alcohol Use Disorder.

**Supplemental Figure 4.**

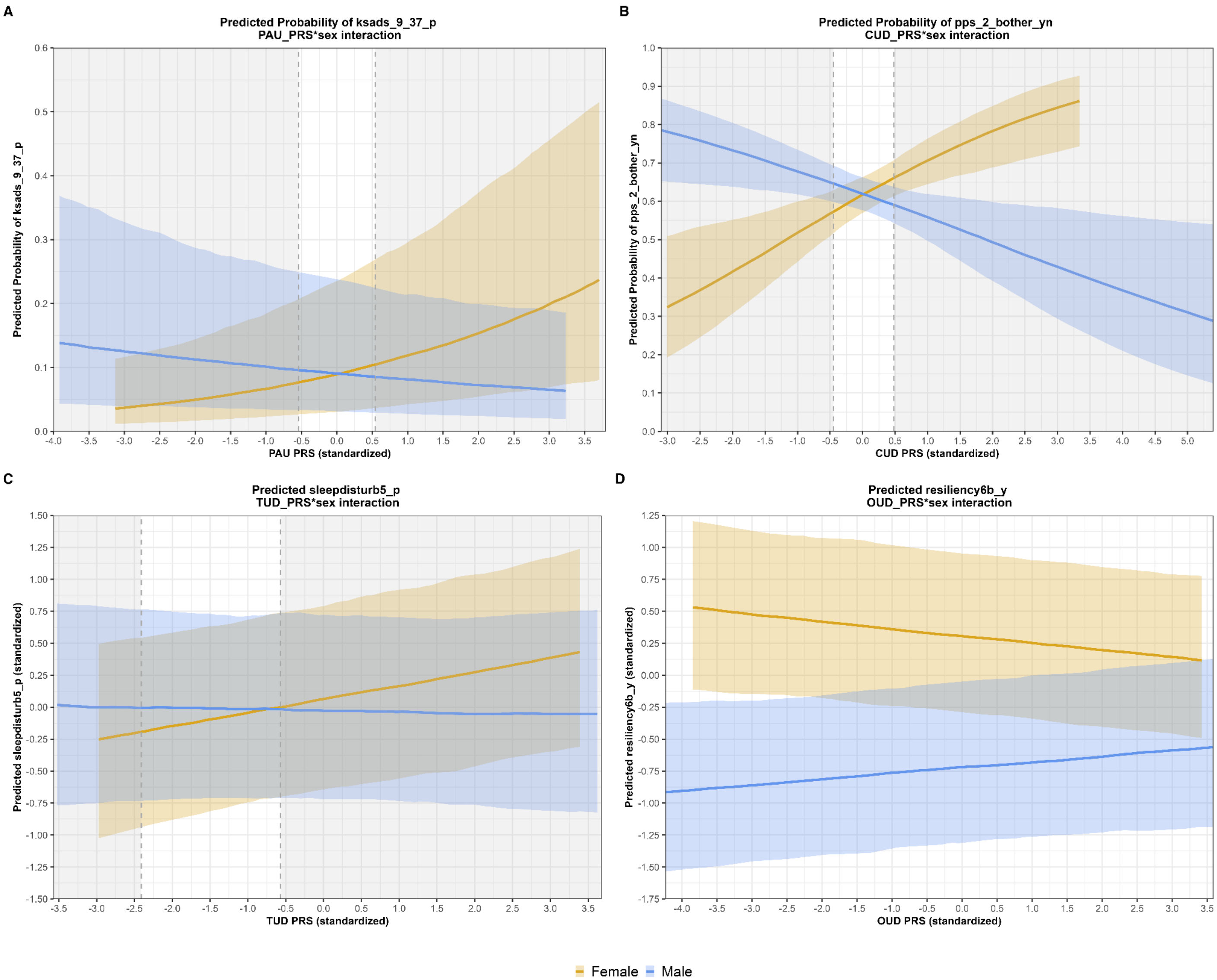


*Note.* Interactions between SUD PGS & biological sex in the sample of participants genetically similar to European reference populations. Confidence intervals around the predicted probabilities are shown in yellow (female) & blue (male). Gray shaded areas indicate Johnson-Neyman intervals for which the interaction is significant. **A.** Interaction between Problematic Alcohol Use (PAU) PGS and biological sex in association with ksads_9_37_p (past active avoidance of a phobic object). Confidence intervals around the predicted probabilities are shown in yellow (female) and blue (male). The simple slope for female participants is statistically significant (B=0.31, *p*=5.12e-05), and the simple slope for male participants is not (B=-0.12, *p*=0.09). The interaction is significant outside of the range -0.54 to 0.54. **B.** Interaction between Cannabis Use Disorder (CUD) PGS and biological sex in association with pps_2_bother_yn (bothered by hearing strange sounds). The simple slopes for female (B=0.42, *p*=2.16e-06) and male (B=-0.24, *p*=0.02) participants are both statistically significant but opposite in direction. The interaction is significant outside of the range of -0.45 to 0.48. **C.** Interaction between Tobacco Use Disorder (TUD) PGS and biological sex in association with sleepdisturb5_p (feeling anxious or disturbed when falling asleep). The simple slope for female participants is statistically significant (B=0.10, *p*=5.63e-06), and the simple slope for male participants is not (B=-0.02, *p*=0.41). The interaction is significant outside of the range of -2.41 to -0.57. **D.** Interaction between Opioid Use Disorder (CUD) PGS and biological sex in association with resiliency6b_y (number of close friends that are girls). The simple slope for male participants is statistically significant (B=0.08, *p*=3.22e-07), and the simple slope for female participants is not (B=-0.02,

*p*=0.26). The Johnson-Neyman Intervals for which the interaction is significant fall outside of the range of observed data and thus are not shown here.

**Supplemental Figure 5.**

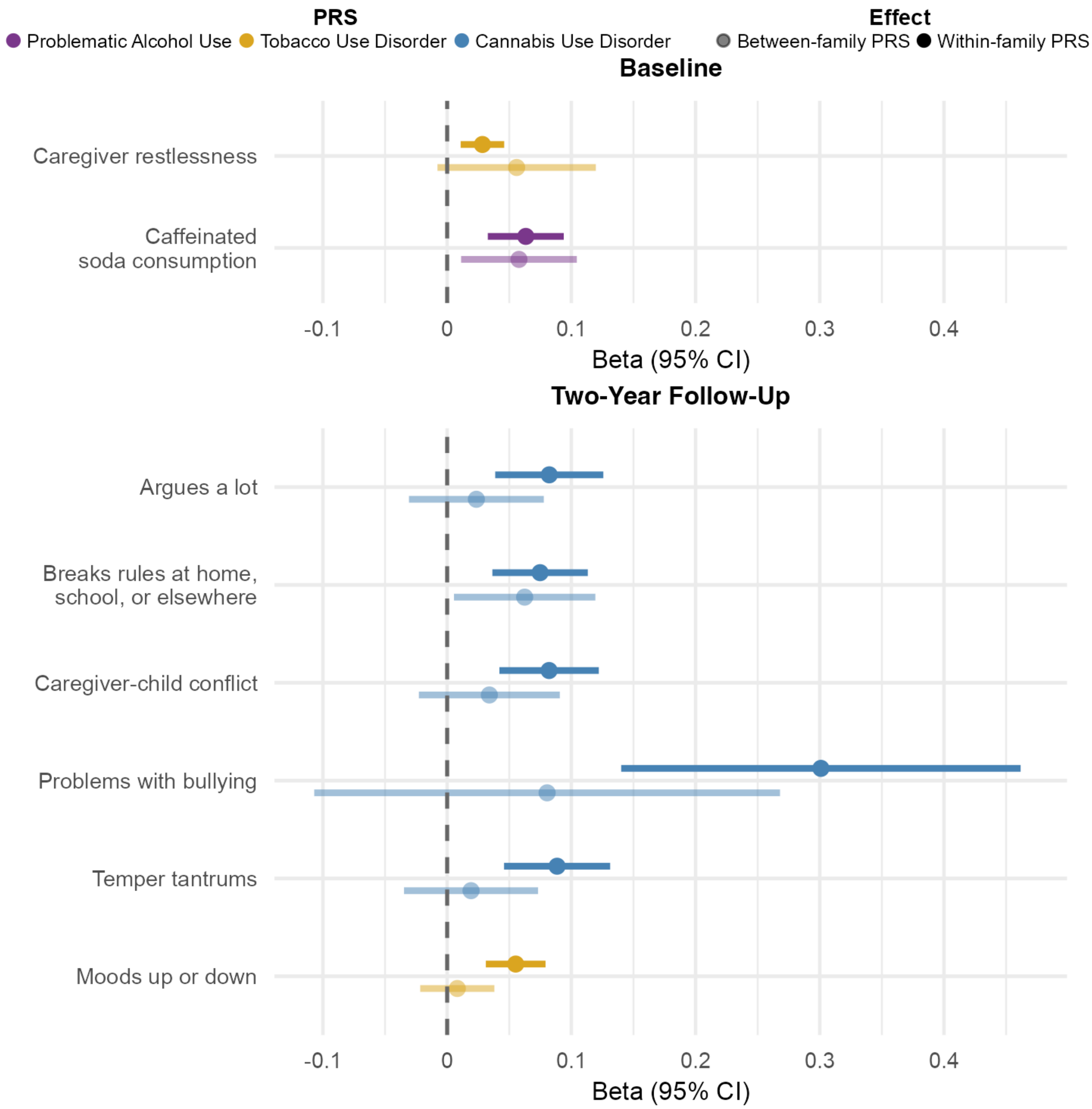


*Note.* Within-family analyses in European-like genetic ancestry. PGS were decomposed into between- and within-family variance and entered into mixed effects models simultaneously as predictors of phenotypes that were significantly associated with the PGS following Bonferroni correction. Depicted here are the Bonferroni-corrected significant within-family PGS associations (in the darker color), alongside the respective between-family PGS effect (lighter color). Point estimates reflect standardized betas, and error bars represent 95% confidence intervals.

**Supplemental Figure 6.**

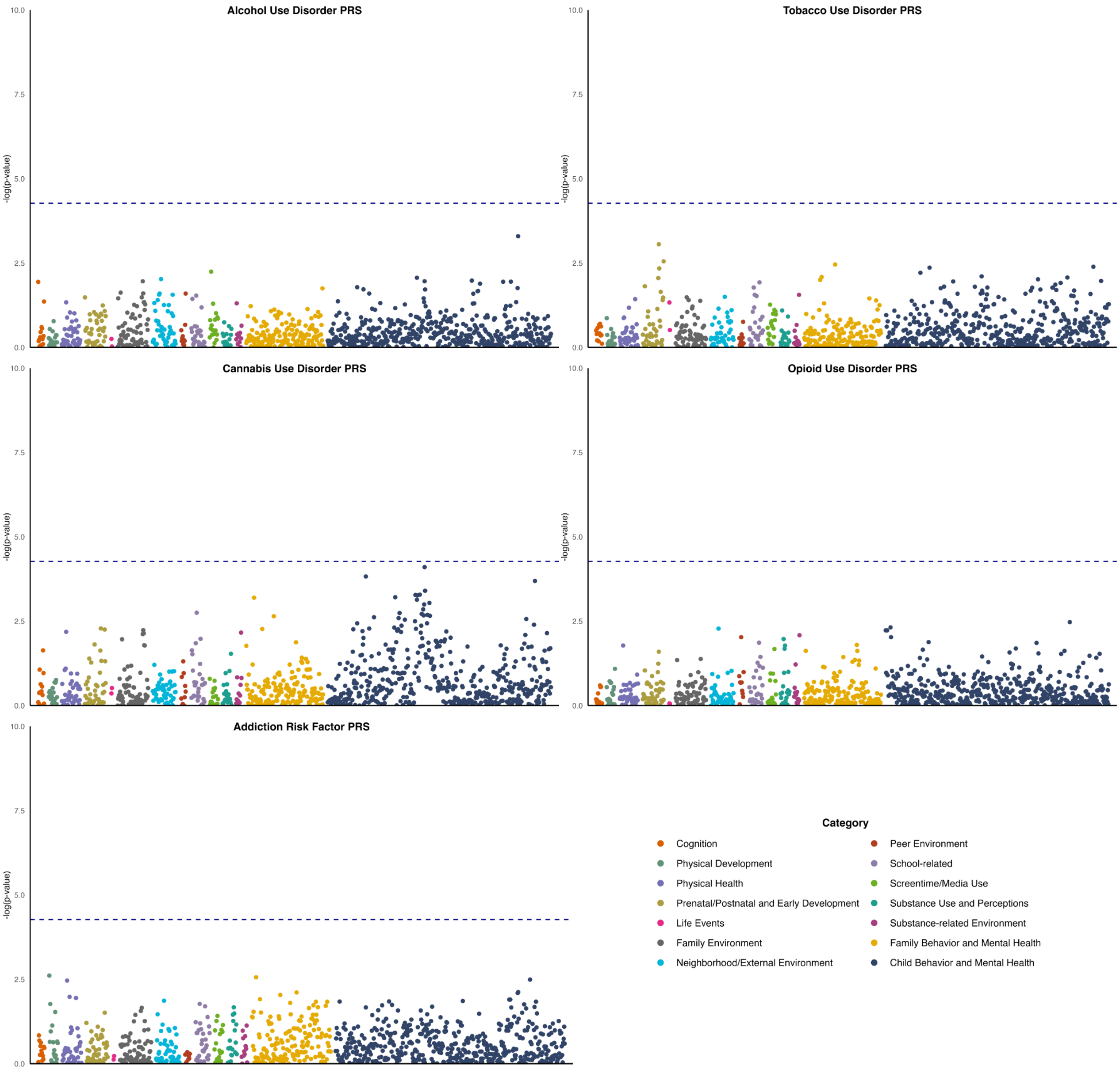


*Note*. Manhattan plots depicting primary PheWAS associations at the baseline wave among individuals genetically similar to African reference populations. The 940 examined phenotypes are spread along the x-axis, and the y-axes represent the -$\log_{10}$ of the uncorrected *p* values of associations between each PGS and each phenotype. The points are color-coded by phenotype domain. The dashed lines indicate the Bonferroni-significant threshold (i.e., -$\log_{10}(0.05/940) = 4.27$). Full results are located in **Supplemental Table 21**.

**Supplemental Figure 7.**

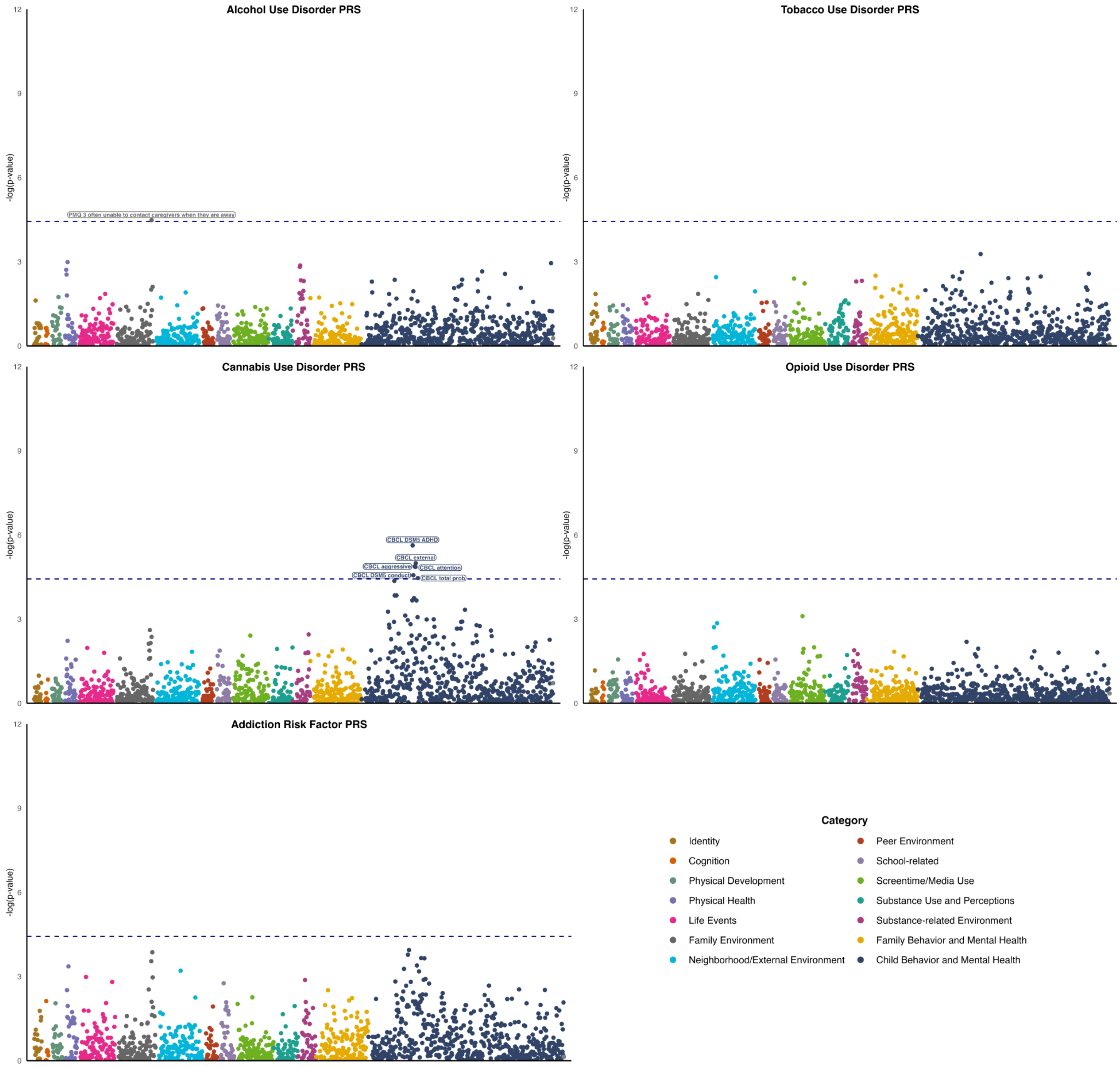


*Note*. Manhattan plots depicting primary PheWAS associations at the two-year follow-up wave among individuals genetically similar to African reference populations. The 1,357 examined phenotypes are spread along the x-axis, and the y-axes represent the $-\log_{10}$ of the uncorrected *p* values of associations between each PGS and each phenotype. The points are color-coded by phenotype domain. The dashed lines indicate the Bonferroni-significant threshold (i.e., $-\log_{10}(0.05/1,357) = 4.43$). Full results are located in **Supplemental Table 22**.

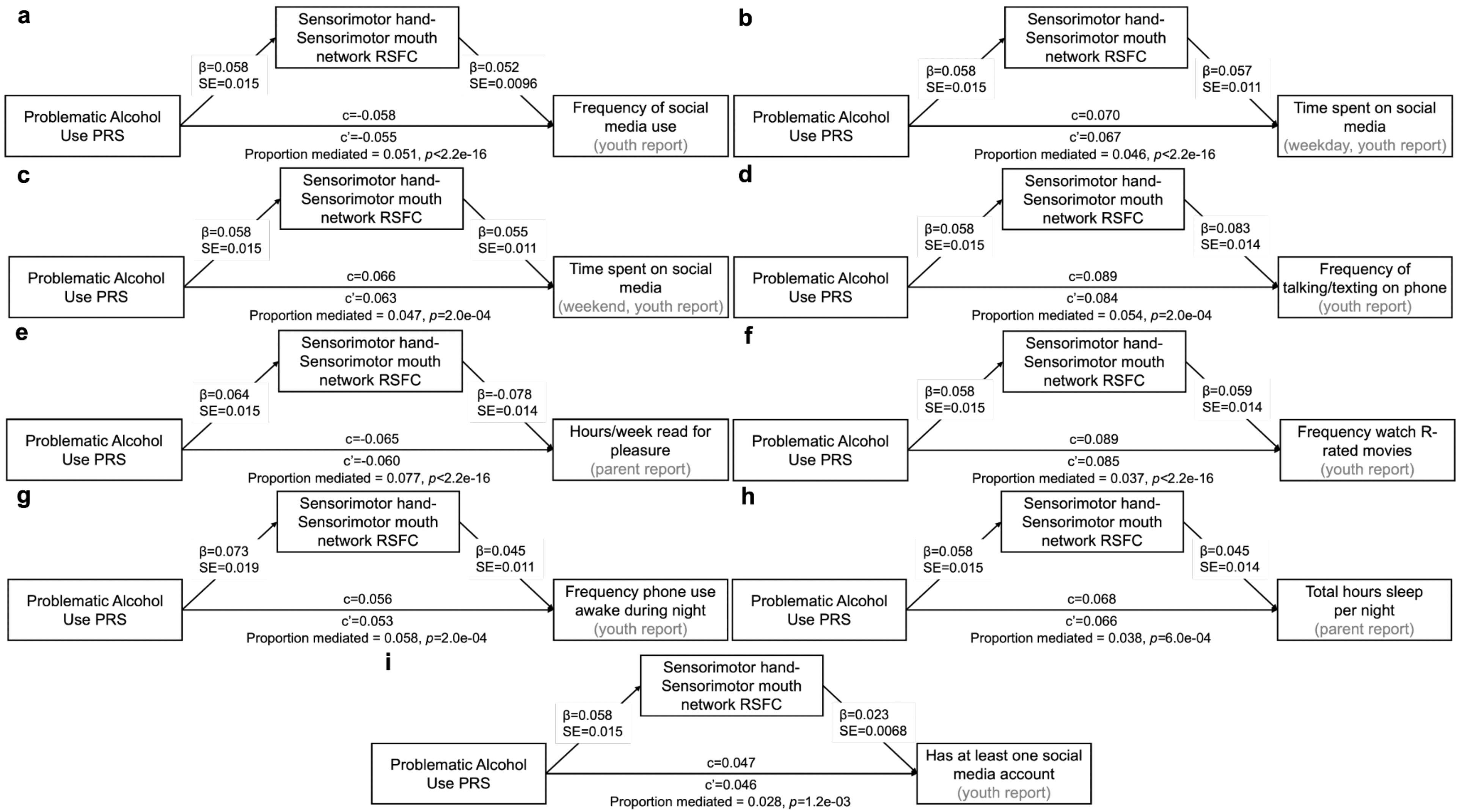


**Supplemental Figure 8.** Indirect effects of Problematic Alcohol Use PGS on screen time, reading, and sleep phenotypes through resting state functional connectivity (RSFC) between the sensorimotor hand and sensorimotor mouth networks. c = total effect; c' = direct

effect. All indirect effects shown are statistically significant following Bonferroni correction. Results are also shown in **Supplemental Table 35** and are from the subsample of individuals genetically similar to European reference populations.

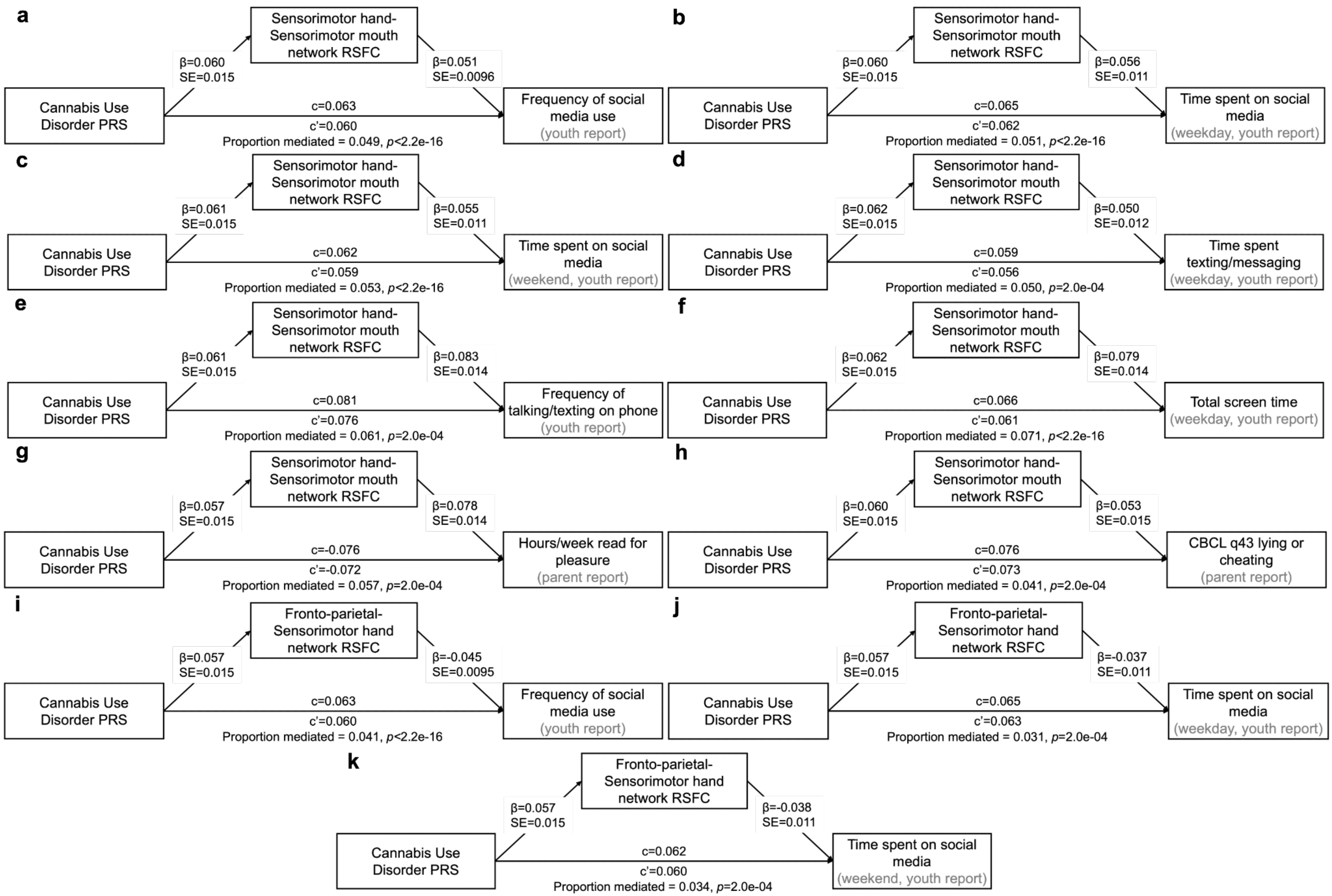
a
Sensorimotor hand-Sensorimotor mouth network RSFC
β=0.060 SE=0.015
β=0.051 SE=0.0096
Cannabis Use Disorder PRS
c=0.063
c'=0.060
Proportion mediated = 0.049, p<2.2e-16
Frequency of social media use (youth report)
b
Sensorimotor hand-Sensorimotor mouth network RSFC
β=0.060 SE=0.015
β=0.056 SE=0.011
Cannabis Use Disorder PRS
c=0.065
c'=0.062
Proportion mediated = 0.051, p<2.2e-16
Time spent on social media (weekday, youth report)
c
Sensorimotor hand-Sensorimotor mouth network RSFC
β=0.061 SE=0.015
β=0.055 SE=0.011
Cannabis Use Disorder PRS
c=0.062
c'=0.059
Proportion mediated = 0.053, p<2.2e-16
Time spent on social media (weekend, youth report)
d
Sensorimotor hand-Sensorimotor mouth network RSFC
β=0.062 SE=0.015
β=0.050 SE=0.012
Cannabis Use Disorder PRS
c=0.059
c'=0.056
Proportion mediated = 0.050, p=2.0e-04
Time spent texting/messaging (weekday, youth report)
e
Sensorimotor hand-Sensorimotor mouth network RSFC
β=0.061 SE=0.015
β=0.083 SE=0.014
Cannabis Use Disorder PRS
c=0.081
c'=0.076
Proportion mediated = 0.061, p=2.0e-04
Frequency of talking/texting on phone (youth report)
f
Sensorimotor hand-Sensorimotor mouth network RSFC
β=0.062 SE=0.015
β=0.079 SE=0.014
Cannabis Use Disorder PRS
c=0.066
c'=0.061
Proportion mediated = 0.071, p<2.2e-16
Total screen time (weekday, youth report)
g
Sensorimotor hand-Sensorimotor mouth network RSFC
β=0.057 SE=0.015
β=0.078 SE=0.014
Cannabis Use Disorder PRS
c=-0.076
c'=-0.072
Proportion mediated = 0.057, p=2.0e-04
Hours/week read for pleasure (parent report)
h
Sensorimotor hand-Sensorimotor mouth network RSFC
β=0.060 SE=0.015
β=0.053 SE=0.015
Cannabis Use Disorder PRS
c=0.076
c'=0.073
Proportion mediated = 0.041, p=2.0e-04
CBCL q43 lying or cheating (parent report)
i
Fronto-parietal-Sensorimotor hand network RSFC
β=0.057 SE=0.015
β=-0.045 SE=0.0095
Cannabis Use Disorder PRS
c=0.063
c'=0.060
Proportion mediated = 0.041, p<2.2e-16
Frequency of social media use (youth report)
j
Fronto-parietal-Sensorimotor hand network RSFC
β=0.057 SE=0.015
β=-0.037 SE=0.011
Cannabis Use Disorder PRS
c=0.065
c'=0.063
Proportion mediated = 0.031, p=2.0e-04
Time spent on social media (weekday, youth report)
k
Fronto-parietal-Sensorimotor hand network RSFC
β=0.057 SE=0.015
β=-0.038 SE=0.011
Cannabis Use Disorder PRS
c=0.062
c'=0.060
Proportion mediated = 0.034, p=2.0e-04
Time spent on social media (weekend, youth report)

**Supplemental Figure 9.** Indirect effects of Cannabis Use Disorder PGS on screen time, reading, and lying/cheating phenotypes through resting state functional connectivity (RSFC) between the sensorimotor hand and sensorimotor mouth networks and between the fronto-parietal and sensorimotor hand networks. c = total effect; c' = direct effect. All indirect effects shown are statistically significant following Bonferroni correction. Results are also shown in **Supplemental Table 35** and are from the subsample of individuals genetically similar to European reference populations.

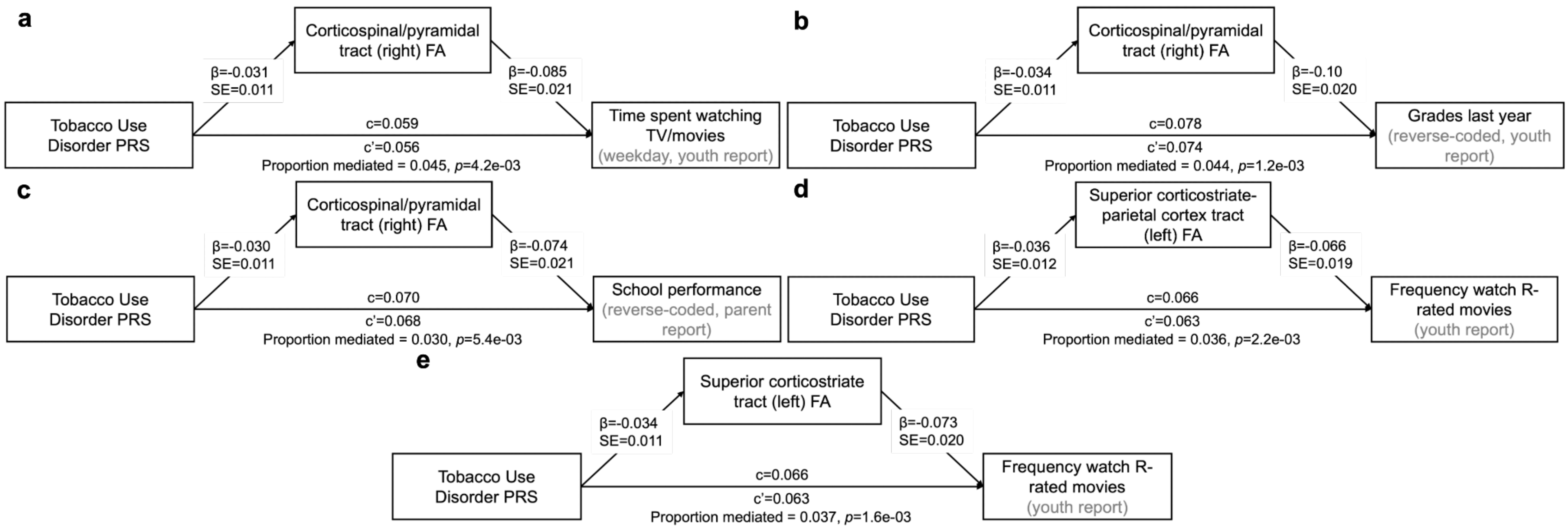


**Supplemental Figure 10.** Indirect effects of Tobacco Use Disorder PGS on screen time and school performance phenotypes through fractional anisotropy in the corticospinal/pyramidal, superior corticostriate-parietal cortex, and superior corticostriate white matter tracts. c = total effect; c' = direct effect. All indirect effects shown are statistically significant following Bonferroni correction. Results are also shown in **Supplemental Table 35** and are from the subsample of individuals genetically similar to European reference populations.
